# Long Non-Coding RNA Profiling in a Non-Alcoholic Fatty Liver Disease Rodent Model: New Insight into Pathogenesis

**DOI:** 10.3390/ijms18010021

**Published:** 2017-01-16

**Authors:** Yi Chen, Haixiu Huang, Chengfu Xu, Chaohui Yu, Youming Li

**Affiliations:** Department of Gastroenterology, The First Affiliated Hospital, College of Medicine, Zhejiang University, 79 Qingchun Road, Hangzhou 310003, China; yiiic@126.com (Y.C.); doctorlc@126.com (H.H.); minkaihu@163.com (C.X.); yiiichenzju@gmail.com (C.Y.)

**Keywords:** long non-coding RNA, non-alcoholic fatty liver disease, circadian rhythm

## Abstract

Non-alcoholic fatty liver disease (NAFLD) is one of the most prevalent chronic liver diseases worldwide with an unclear mechanism. Long non-coding RNAs (lncRNAs) have recently emerged as important regulatory molecules. To better understand NAFLD pathogenesis, lncRNA and messenger RNA (mRNA) microarrays were conducted in an NAFLD rodent model. Potential target genes of significantly changed lncRNA were predicted using *cis*/*trans*-regulatory algorithms. Gene Ontology (GO) analysis and Kyoto Encyclopedia of Genes and Genomes (KEGG) pathway enrichment analysis were then performed to explore their function. In the current analysis, 89 upregulated and 177 downregulated mRNAs were identified, together with 291 deregulated lncRNAs. Bioinformatic analysis of these RNAs has categorized these RNAs into pathways including arachidonic acid metabolism, circadian rhythm, linoleic acid metabolism, peroxisome proliferator-activated receptor (PPAR) signaling pathway, sphingolipid metabolism, steroid biosynthesis, tryptophan metabolism and tyrosine metabolism were compromised. Quantitative polymerase chain reaction (qPCR) of representative nine mRNAs and eight lncRNAs (named fatty liver-related lncRNA, FLRL) was conducted and this verified previous microarray results. Several lncRNAs, such as FLRL1, FLRL6 and FLRL2 demonstrated to be involved in circadian rhythm targeting period circadian clock 3 (Per3), Per2 and aryl hydrocarbon receptor nuclear translocator-like (Arntl), respectively. While FLRL8, FLRL3 and FLRL7 showed a potential role in PPAR signaling pathway through interaction with fatty acid binding protein 5 (Fabp5), lipoprotein lipase (Lpl) and fatty acid desaturase 2 (Fads2). Functional experiments showed that interfering of lncRNA FLRL2 expression affected the expression of predicted target, circadian rhythm gene *Arntl*. Moreover, both FLRL2 and Arntl were downregulated in the NAFLD cellular model. The current study identified lncRNA and corresponding mRNA in NAFLD, providing new insight into the pathogenesis of NAFLD. Moreover, we identified a new lncRNA FLRL2, that might participate NAFLD pathogenesis mediated by Arntl.

## 1. Introduction

Non-alcoholic fatty liver disease (NAFLD) is one of the most prevalent chronic liver diseases worldwide, affecting approximately 20% of the general population and up to about 70% in patients with type 2 diabetes [1]. It is widely accepted that NAFLD consists of four histological stages, namely simple steatosis, non-alcoholic steatohepatitis (NASH), fibrosis and cirrhosis [2]. Simple steatosis is recognized as benign liver disease with slow exacerbation over decades, whereas NASH may progress into cirrhosis and even hepatocellular carcinoma in a short period of time [3]. Thus, comprehensive understanding of NAFLD pathogenesis is of supreme importance concerning treatment optimization and disease prevention.

Pathogenesis of NAFLD is complicated. The most widespread and prevailing hypothesis is the so-called “two-hit” model [4]. The first hit results from insulin resistant accompanied by fat accumulation and then on this basis, oxidative stress induces varied inflammatory cytokines (tumor necrosis factor-α (TNF-α), interleukin (IL) 1, IL-6, etc.) as well as adipokines (leptin, adiponectin and resistin), resulting in hepatic inflammation and hepatocyte injury, known as the second hit. Current management of NAFLD included lifestyle modification, but with poor compliance. Thus, medical intervention is necessary, such as insulin sensitizer, lipid lowering agents, and antioxidants, which incur a prolonged high cost [5].

Long non-coding RNAs (lncRNAs) are RNA transcripts longer than 200 nucleotides, without proteins translation capacity [6]. LncRNA could either act as transcriptional regulators by mediating gene activation or silencing through chromatin modification [7]. Post-transcriptional regulation of lncRNA included base pairing of messenger RNA (mRNA) and using decoys of RNA-binding proteins/microRNAs (miRNAs) to suppress splicing [8]. LncRNAs are involved in numerous cellular processes, such as cell growth, differentiation, apoptosis [9] and cancer metastasis [10].

LncRNAs are becoming increasingly focused on pathogenesis of liver diseases and have been shown to have potential diagnostic, prognostic, and therapeutic importance [11]. H19 is among the earliest identified and widely investigated lncRNAs in liver disease. H19 was elevated in hepatocellular carcinoma and suppressed tumor metastases in a miR-220-dependent way [12]. Moreover, lncRNAs, including HULC [13], MALAT-1 [14] and HOTAIR [15] were found to serve as biomarker of liver cancer prognosis and are associated with a response to chemotherapy as well. In a recent report, MALAT-1 has also been shown to be related with metabolic disorder [16]. In addition to hepatocellular carcinoma, lncRNAs also participated in liver lipid metabolism [17], hepatitis B virus (HBV) infection [18] and HELLP (i.e., hemolysis, elevated liver enzymes, low platelets) syndrome [19].

In this study, we performed a genome-wide lncRNA and mRNA microarray analysis of liver samples from NAFLD rodent model, in order to identify valuable RNAs in hepatosteatosis, thus expanding understanding of NAFLD pathogenesis and to provide new insights for the development of new therapeutic approaches.

## 2. Results

### 2.1. Animal Model of Non-Alcoholic Fatty Liver Disease

To investigate the potential role of lncRNA in NAFLD, microarray analysis in liver tissue of four high fat diet mice and four chow diet control mice was conducted. NAFLD mice develop significant hepatic steatosis (Figure S1).

### 2.2. Long Non-Coding RNA Profile in Non-Alcoholic Fatty Liver Disease

**Figure 1:**
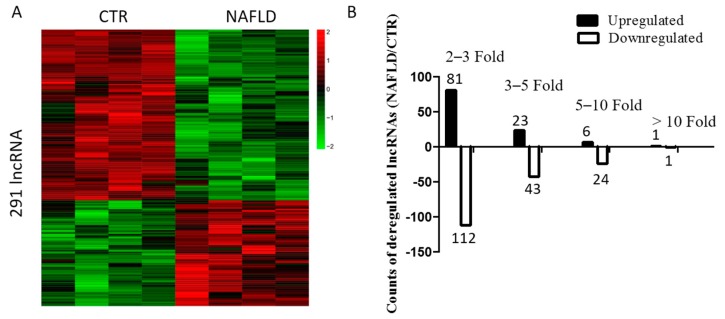
Long non-coding RNAs (lncRNA) profile in non-alcoholic fatty liver disease (NAFLD). (**A**) Heat map of differentially expressed lncRNAs in control (CTR) and NAFLD mice. Each row shows relative expression levels of a single lncRNA and each column represents expression levels for a single sample. Relative high (red) or low (green) expression is indicated; (**B**) Expression profiling of lncRNAs in NAFLD and control mice. Up- or downregulated lncRNAs were defined as normalized probe signal intensity of lncRNAs that changed more than two-fold in NAFLD compared with control. Black bars represent the number of upregulated lncRNAs, whereas white bars represent the number of downregulated lncRNAs.

Using microarray analysis, 291 lncRNAs were found to be deregulated in NAFLD (Figure 1A, Table S1), 111 lncRNAs were upregulated and 180 lncRNAs were downregulated (Figure 1B).

### 2.3. Bioinformatic Analysis and Characterization of Differentially Expressed lncRNAs

Potential targets of these 291 lncRNA were predicted using University of California Santa Cruz (UCSC) genome browser and BLAST as described in the Materials and Methods section. A total of 12,606 *cis*-targets and 70,574 *trans*-targets were identified and adopted as surrogates of lncRNA in further functional analysis (Tables S2 and S3). To help interpret the biological function of these targets and indeed the altered lncRNA profiles, Gene Ontology (GO) and Kyoto Encyclopedia of Genes and Genomes (KEGG) pathway analysis were performed.

GO analysis included three categories of biological function, namely “biological process”, “cellular component” and “molecular function” (Table S4). All targets were divided into four groups, defined by whether they were *cis* or *trans*, and whether their lncRNAs were upregulated or downregulated. Among all biological processes, “RNA polymerase II promoter”, “rhythmic process” and “fatty acid metabolism process” were the three most changed biological processes in NAFLD in both *cis*-targets of up and downregulated lncRNAs and “translation”, “carboxylic acid transport” and “organic acid transport” were the most changed for the *trans*-targets of all lncRNAs. The three most changed “cellular components” among *trans*-targets of downregulated lncRNAs were “non-membrane-bound organelle”, “intracellular non-membrane-bound organelle” and “condensed chromosome, centrometric region”. Molecular function GO analysis of both *cis*-targets categories revealed “oxidoreductase activity” as the most significantly changed one. As for those *trans*-targets of both up- and downregulated lncRNAs, the three most changed were “transition metal ion binding”, “cation binding” and “metal ion binding”.

To gain further insights into pathogenesis of NAFLD, signaling pathway enrichment analysis was done with the KEGG database. Unlike GO analysis, different groups of targets showed distinct pathway enrichment patterns (Table S1). “Circadian rhythm” and “peroxisome proliferator-activated receptor (PPAR) signaling pathway” were the most significantly enriched pathways in *cis*-targets of upregulated lncRNAs. Pathway analysis of *cis*-targets of downregulated lncRNAs showed significant enrichment in “glycerolipid metabolism”. As for *trans*-targets, “apoptosis”, “circadian rhythm” and “sphingolipid metabolism” were identified in elevated lncNRAs and “steroid biosynthesis”, “vascular endothelial growth factor (VEGF) signaling pathway” and “alanine, aspartate and glutamate metabolism” were identified in targets of downregulated lncRNAs. Table S2 provides examples of lncRNAs and their predicted target pathway.

### 2.4. Global Changes in Gene Expression Profiles of Non-Alcoholic Fatty Liver Disease and Bioinformatic Analysis

**Figure 2:**
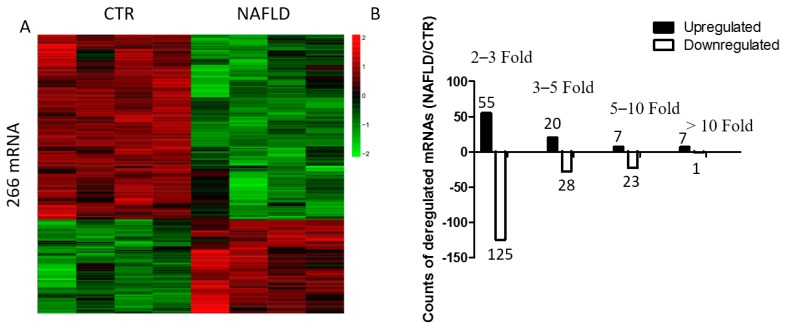
Microarray result of mRNA profile in NAFLD. (**A**) Heat map of differentially expressed mRNAs in control and NAFLD mice. Each row shows relative levels of each mRNA expression and each column represents expression levels for a single sample. Relative high (red) or low (green) expression is indicated; (**B**) Expression profiling of mRNAs in NAFLD and control mice. Determination of upregulated and downregulated mRNAs was based on more than two-fold changes of the normalized probe signal intensity in NAFLD compared with control. Black bars represent the number of upregulated mRNAs, whereas white bars represent the number of downregulated mRNAs.

To further evaluate transcriptional regulation in NAFLD, gene expression profiling was performed using Agilent mouse lncRNA 4 × 180 K microarray. Two hundred and sixty-six differentially expressed genes were identified, of which 89 were upregulated and 177 downregulated (Figure 2, Table S5).

In GO analysis, “oxidation reduction”, “microsome”, and “glutathione transferase activity” were found associated with NAFLD (Table S6). In pathway analysis, significant transcriptional changes of several canonical signaling pathways in the KEGG database were revealed in NAFLD (Table S3). These signaling pathways included “metabolism of xenobiotics by cytochrome P450”, “metabolism of drug, retinol, arachidonic acid, glutathione, fatty acid and steroid” as well as “PPAR signaling pathway” and “circadian rhythm” for upregulated genes. Similar results were found for downregulated genes.

### 2.5. Quantitative Polymerase Chain Reaction Validation of Microarray Data

**Figure 3:**
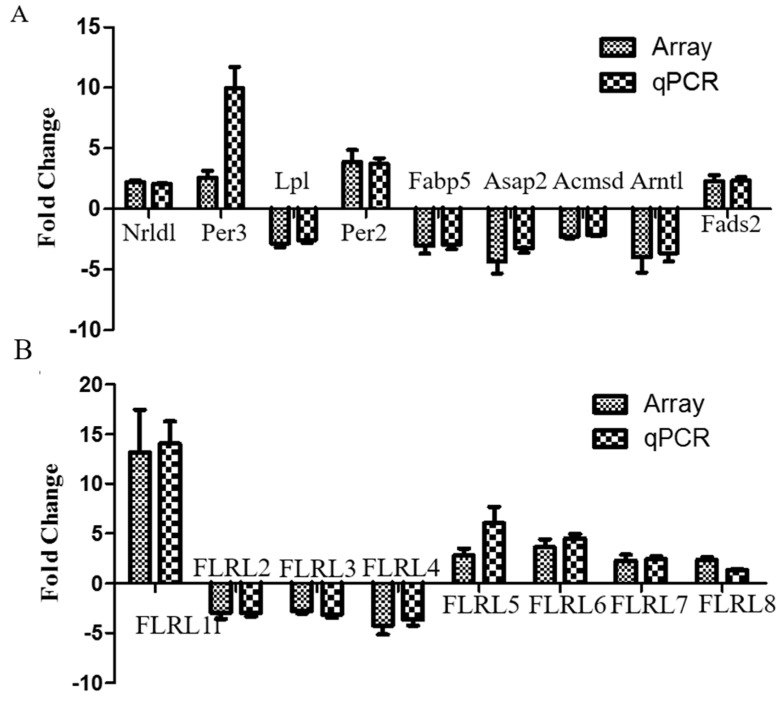
Fold change in mRNA (**A**) and lncRNA levels (**B**) inNAFLD compared with control mice, by microarray and quantitative polymerase chain reaction (qPCR) analyses. Bars above the *x*-axis indicat that genes are upregulated, and bars below the *x*-axis indicat that genes are downregulated. Data are presented as the mean ± standard deviation (SD). No significant differences were found for any genes assessed either by array or qPCR.Nr1d1, nuclear receptor subfamily 1, group D, member 1; Per3, period circadian clock 3; Lpl, lipoprotein lipase; Per2, period circadian clock 2; Fabp5, fatty acid-binding protein 5; Asap2, Acmsd, Arf-GAP with SH3 domain, ankyrin repeat and PH domain 2; Arntl, aryl hydrocarbon receptor nuclear translocator-like; Fads2, fatty acid desaturase; FLRL, fatty liver related lncRNA.

To confirm microarray results, nine mRNAs and eight lncRNAs (named fatty liver related lncRNA, FLRL) that were involved in NAFLD-related pathways, including circadian rhythm, PPAR signaling and tryptophan metabolism, were selected for qPCR analysis. As expected, expression patterns of these genes were consistent with the microarray data (Figure 3), ensuring reliability of the microarray assay.

### 2.6. Common Pathway of Different Expressed lncRNA and Corresponding mRNA

**Figure 4:**
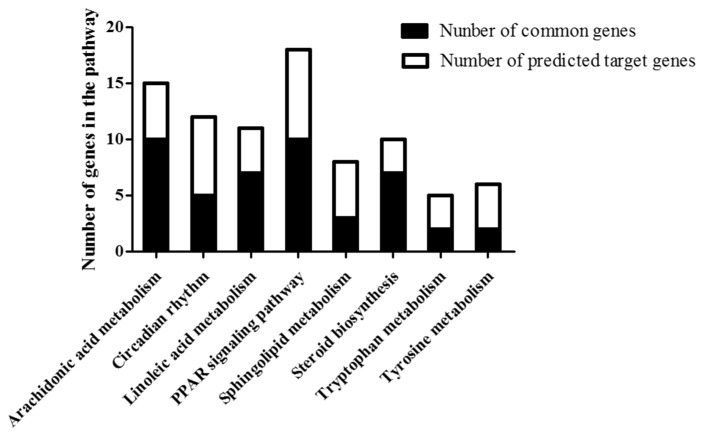
Pathway interplay analysis of lncRNA and mRNA. Genes which are both potential targets of differentially expressed lncRNAs and belong to differentially expressed mRNAs were identified. Black bars indicate the number of intersecting target genes located in the pathway, whereas white bars indicate the number of predicted lncRNA target genes falling in each pathway. PPAR, Peroxisome proliferator-activated receptor.

To exclude false negative or false positive results, interplay between target genes of lncRNA and genes identified by mRNA microarray were analyzed. Interestingly, several signaling pathways, such as arachidonic acid metabolism, circadian rhythm, linoleic acid metabolism, PPAR signaling pathway, sphingolipid metabolism, steroid biosynthesis, tryptophan metabolism and tyrosine metabolism were significantly changed in NAFLD in both lncRNA target analysis and mRNA enrichment analysis (Figure 4). These eight pathways included 39 predicted lncRNA targets and 46 mRNAs and 39 of them overlapped. Interestingly, among all 39 predicted lncRNA targets, four from circadian rhythm pathway and three from PPAR signaling pathway are *cis*, and the rest are *trans*. As for these 39 targets, 35 changed in the same direction as their lncRNA, and four were conversely regulated compared with their lncRNA. However, target prediction did not show the direction of these changes, which means that each lncRNA can have either an inhibitory or a promotive effect on its targets, whether in *trans* or *cis*. It appears that most of the lncRNA analyzed here tend to regulate their target genes positively. So, concerns emerged that the overlapping of these pathways may be caused by the regulatory effect of changed lncRNAs targeting certain mRNAs.

### 2.7. Knockdown of lncRNA FLRL2 Increases Predicted-Target Arntl Expression and Overexpression Vice Versa

**Figure 5:**
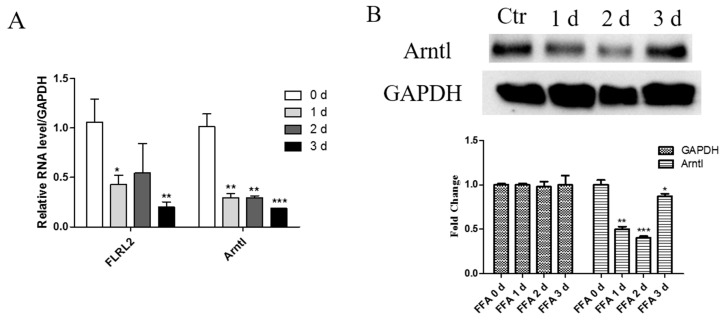
Decreased expression of fatty liver related lncRNA 2 (FLRL2) and aryl hydrocarbon receptor nuclear translocator-like (Arntl) in NAFLD cellular model. AML12 cells were treated with 1 mM free fatty acids (FFAs) for 1, 2, and 3 days (d) or 5% bovine serum albumin (BSA) as control. Cell extracts of total RNA (**A**) and protein (**B**) were collected and then tested accordingly. Expression of Arntl and FLRL2 were normalized to glyceraldehyde 3-phosphate dehydrogenase (GAPDH) in qPCR analysis. Data are presented as the mean ± SD.* *p* < 0.05; ** *p* < 0.01; *** *p* < 0.001.

To further support animal data, an NAFLD cellular model was constructed with free fatty acid (FFA) treatment and verified with Oil Red O staining, as well as cellular triglyceride (TG) quantification (Figure 5). As FFA was treated for longer, the cellular lipid, stained with red dye, was more obvious, and steatosis was gradually aggravated in a time-dependent manner, proved by progressively elevated TG levels (Figure S2). In a NAFLD cellular model, consistent with the in vivo study, the expression of both Arntl and FLRL2 was inhibited (Figure 5).

**Figure 6:**
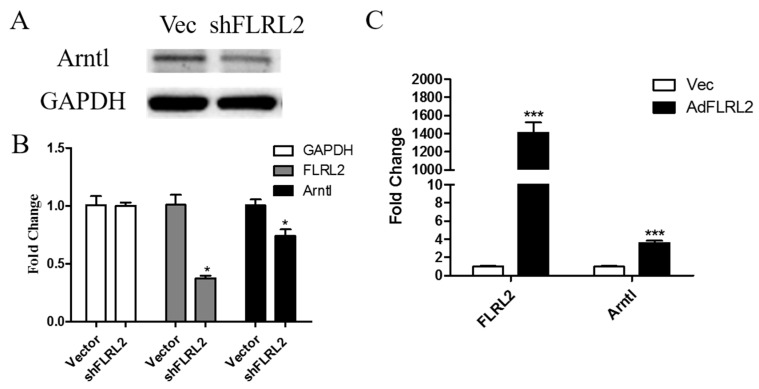
FLRL2 knockdown downregulates predicted target *Arntl* expression and vice versa. shFLRL2 plasmids and empty vectors as a control were transfected in AML12 cells for 48 h. Cell extracts were prepared for Western blot (**A**) and qPCR (**B**); FLRL2 overexpression vector, AdFLRL2 plasmid was transfected and total RNA were extracted after 48 h (**C**). mRNA levels of FLRL2 and Arntl were measured by qPCR and presented as the mean ± SD relative to the levels of control cells from three experiments. * *p* < 0.05; *** *p* < 0.001.

In order to provide evidence for validity of lncRNA target prediction, and to further explore the role of FLRL2 in NAFLD, knockdown and overexpression of FLRL2 was performed in AML12 cells. In order to limit off-target effect in the knockdown study, a total of three short hairpin RNAs (shRNAs) against FLRL2 (shFLRL2-1, shFLRL2-2, shFLRL23) were adopted, and sh2 (short for shFLRL2-2) was finally adopted in a further study for the most prevalent inhibiting effect (Figure S3). Western blot and qPCR results showed that knockdown of FLRL2 led to *Arntl* downregulation, and in contrast, overexpression causes Arntl mRNA elevation, indicating a positive regulatory role of FLRL2 in Arntl (Figure 6). To further support the inhibitory effect of FLRL2 on Arntl enpression, experiment with a modified FLRL2 transcript that is not targeted by the shRNA was conducted and verified our hypothesis (Figure S4).

## 3. Discussion

Increasing evidence has revealed that lncRNAs play an important role in gene expression control [20]. Although thousands of lncRNAs have been identified in recent years, lncRNA profiling in metabolic diseases, such as NAFLD, has not been reported yet. This study was focused on lncRNA expression spectrum in an NAFLD rodent model, together with mRNA, in order to elucidate the molecular mechanisms underlying pathogenesis of NAFLD. Microarray analysis revealed 266 differentially expressed genes, with 89 upregulated and 177 downregulated, together with 291 deregulated lncRNAs, with 111 increased and 180 decreased. Among all 291 deregulated lncRNAs, 19.9% have homologs between mice and humans. Notably, a previous study reported a global expression of lncRNA in NAFLD patients, which showed a different profile compared with ours [21]. Although the human lncRNA profile brought more direct data, while the mouse model just acted as a surrogate, there are certain limitations. Firstly, there has been huge development in diagnostic approaches of NAFLD, including an imaging study (ultrasound, magnetic resonance imaging (MRI), etc.) and serum biomarker analysis [22]. Although it is still the golden standard, liver pathology is invasive, so patients with simple steatosis do not routinely receive a live biopsy. Although liver samples from pure NAFLD patients versus normal control would be the best to clarify lncRNA profiling under this circumstance, it is neither easy nor to ethically sound to achieve that. On the other hand, use of liver samples from other diseases, such as gallbladder stone patients instead, might to some extent complicate this context. Secondly, as for humans, many factors such as education, environment, life style might affect epigenomes in each individual. Thus, in reality, a great variety exists, and false positive results may be taken into consideration in lncRNA profiling in NAFLD [13,23]. High fat diet-fed mice were a mature NAFLD animal model with favorable pathological stability and similar genetic background and our group possess a sound technique and great experience in constructing this model [24,25,26]. Therefore, herein, we adopted NAFLD mice model rather than human samples in lncRNA profiling.

To better understand lncRNA profile in NAFLD, targets of lncRNA were predicted and informatic analysis, such as GO analysis and pathway analysis were conducted. Among all pathways included, arachidonic acid metabolism, circadian rhythm, linoleic acid metabolism, PPAR signaling pathway, sphingolipid metabolism, steroid biosynthesis, tryptophan metabolism, and tyrosine metabolism were identified as common pathways. In addition, there are several other lncRNA association research models [23]. These study models were mainly divided into two groups, including computational models, such as HyperGeometric distribution for LncRNA-Disease Association inference (HGLDA) [24], Fuzzy Measure-based LNCRNA functional SIMilaritycalculation model [25], Improved Random Walk with Restart for LncRNA-Disease Association prediction [26], Improved LNCRNA functional SIMilarity calculation model [27], etc., and other biological network-based models as well. With these biological and computational models, functions of lncRNA in NAFLD would be better interpreted. The current analysis adopted the most preliminary and accessible analysis, RNAplex. Further study should focus on deep investigation of lncRNA in NAFLD with these new approaches.

Notably, five mRNAs and seven lncRNAs related to circadian rhythm changed their expression significantly in NAFLD. Circadian rhythm is defined as endogenous fluctuations of biochemical, physiological, and behavioral activities. It was modulated by a pacemaker entity synchronized by environmental cues [22]. *Per2*, *Per1*, *Arntl*, cryptochrome circadian clock 1 (*Cry1*), *Cry2* and nuclear receptor subfamily 1, group D, member 1 (*Nr1d/1l*) are called “clock genes”, forming network of circadian regulation system [28]. In pathway analysis, lncRNA FLRL6 targeting key element of circadian rhythm Per2 was found to be 3.3-fold increased, consistent with which, Per2 mRNA level was 3.5-fold upregulated, indicating a positive regulation pattern. Previous study in white adipocyte tissue revealed that Per2 could interact with PPARγ by blocking the recruitment of target promoter and subsequently repressingits transcriptional activity and resulting in a pro-adipogetic effect, which indicated a potential role of Per2 in hepatic lipid metabolism [29]. Furthermore, *Arntl*-deficient mice developed obesity with increased food intake, reduced energy expenditure and decreased level of polyunsaturated fatty acid in both adipocytes and serum, displaying destroyed energy hemostasis [30]. In liver, the lack of Arntl reduced fat storage capacity in adipose tissue, resulting in an increase in levels of circulating fatty acids, including triglycerides, free fatty acids, and cholesterol, including ectopic fat formation in the liver [31]. Consistently, lncRNA FLRL2, assumed upstream lncRNA of Arntl, was reported to be three-fold downregulated, and, in addition, Arntl mRNA level was three-fold decreased, predicting a positive regulating role of FLRL2 in *Arntl* expression. Knockdown and overexpression experiment further supported this hypothesis. In order to limit the off-target effect in knockdown study, we adopted three FLRL2 shRNAs, and did a rescue experiment. Despite this, the off-target effect still existed, and further experiments would focus on interaction between FLRL2 and *Arntl* gene, through chromatin immunoprecipitation (ChIP), for example.

Increasing evidence linked lncRNA to lipid metabolism. LncRNA, liver-specific triglyceride regulator, was recently identified and showed to inhibit apolipoprotein C2 (apoC2) expression through a famesoid X receptor-mediated pathway, whose depletion led to robust lipoprotein lipase activation and hypertriglyceridemia [17]. Furthermore, HULC, a long non-coding RNA overexpressed in hepatocellular carcinoma, modulated dysregulation of lipid metabolism in hepatocellular carcinoma by activating acyl-CoA synthetase subunit 1, promoting lipogenesis and thereby stimulating accumulation of intracellular triglycerides and cholesterol [32]. In this study, several lncRNAs were identified to be related to lipogenesis, such as FLRL8, FLRL3 and FLRL7, through proteins in PPAR signaling pathway, such as Fabp5, Lpl and Fads2, indicating their potential regulatory role in lipid metabolism.

Another major finding of this study was that seven mRNAs and five lncRNAs related to linoleic acid metabolism were significantly altered in NAFLD. Linoleic acid, a collection of octadecadienoic fatty acid isomers, have been shown to be beneficial to health by reducing adiposity, anti-diabetogenic, anti-atherogenic, and anti-carcinogenesis [33]. Studies in linoleic acid-fed fish revealed that linoleic acid promoted fish growth, and increased lipid concentration in the whole body and muscle, by promoting transcription of genes related to fatty acid oxidation (carnitine palmitoyl transferase I and acyl-CoA oxidase) and inhibiting PPARα/acyl-CoA oxidase expression [34]. Further studies are needed to investigate the molecular mechanism of linoleic acid in NAFLD pathogenesis.

## 4. Materials and Methods

### 4.1. Animal Model of Non-Alcoholic Fatty Liver Disease and Liver Samples

Male C57BL/6J mice (Experimental Animal Center of Zhejiang Province, Hangzhou, China) were fed with chow diet or high-fat diet (HFD) (60% fat; D12492; Research diet, New Brunswick, NJ, USA) for 8 weeks (*n* = 4 per group) [35]. Mice were euthanized 8 weeks after being placed on the respective treatment and liver samples were acquired. All experiments were conducted with the approval of the First Affiliated Hospital of Zhejiang University Institutional Review Board (Protocol SYXK2013-0180) and in accordance with certain guidelines and in accordance with the Animals (Scientific Procedures) Act 1986 and associated guidelines [36]. Intracellular triglyceride contents were detected using a triglycerides assay kit (Applgyen Technologies Inc., Beijing, China) according to the manufacturer’s recommended protocols. Study design and procedure is shown in Figure S5 as a flowchart.

### 4.2. RNA Extraction and Purification

Total RNA was extracted using TRIzol reagent (Life Technologies, Carlsbad, CA, USA) and checked for a RNA integrity number (RIN) number using an Agilent Bio-analyzer 2100 (Agilent Technologies, Santa Clara, CA, USA). Qualified total RNA was further purified by RNeasy mini kit (QIAGEN, GmBH, Hilden, Germany) and RNase-Free DNase Set (QIAGEN, GmBH).

### 4.3. LncRNA and mRNA Microarray

Agilent Mouse lncRNA 4 × 180 K microarray (design ID 046161, Agilent Technologies, Santa Clara, CA, USA, including 22,231 lncRNAs and 39,430 mRNAs) was used to identify different expressed lncRNA and mRNA in NAFLD animal models. Procedures were described as follows.

Total RNA was amplified and labeled by Low Input Quick Amp Labeling Kit, One-Color (Agilent Technologies, Santa Clara, CA, USA). Labeled cRNA (compliment RNA) were purified by RNeasy mini kit (QIAGEN, GmBH).

Each Slide was hybridized with 1.65 μg cyanine 3 (Cy3)-labeled cRNA using Gene Expression Hybridization Kit (Agilent technologies) in Hybridization Oven (Agilent Technologies). After 17 h hybridization, slides were washed in staining dishes (Thermo Shandon, Waltham, MA, USA) with Gene Expression Wash Buffer Kit (Agilent Technologies).

Slides were scanned by Agilent Microarray Scanner (Agilent technologies). Data were extracted with Feature Extraction software 10.7 (Agilent Technologies). Raw data were normalized by Quantile algorithm, Gene Spring Software 11.0 (Agilent Technologies).

### 4.4. Quantitative Polymerase Chain Reaction Validation

Microarray data were validated by qPCR using SYBR Green Real-Time PCR Master Mix reagents (Toyobo, Osaka, Japan). Primers were designed based on cDNA sequence (Table S7) using Primer Premier 5 (PREMIER Biosoft Int., Palo Alto, CA, USA). Actin was used as a control.

### 4.5. Cell Culture, Non-Alcoholic Fatty Liver Disease Cellular Model and Plasmid Transfection

AML12 cells were cultured in Dulbecco’s modified Eagle’s medium (ThermoFisher Scientific Inc., Cleveland, OH, USA) supplemented with 10% fetal bovine serum (ThermoFisher Scientific Inc., Cleveland, OH, USA) at 37 °C under 5% CO_2_. AML12 cells were exposed to mixture of 1 mM FFAs (0.333 mM oleic acid and 0.667 mM palmitic acid) for 1, 2, or 3 days, to induce steatosis of different degree. Stock solutions of 20 mM oleate and 20 mM palmitate prepared in culture medium containing 5% BSA were conveniently diluted in culture medium to obtain the desired final concentrations. Transient transfections were performed using Lipofectamine 3000 according to the manufacturer’s protocol (Invitrogen, Cleveland, OH, USA).

### 4.6. Protein Extraction and Western Blot

Cell lysate was harvested at 48 h after transfection. Protein from lysed cells was resolved on sodium dodecyl sulfate–polyacrylamide gels and then transferred to a polyvinylidene difluoride membrane. After 5% (*w*/*v*) BSA treatment as blocking in Tris-buffered saline tween (150 mM NaCl, 10 mM Tris-HCl, pH 7.5 and 0.1% Tween 20), corresponding primary and secondary antibodies were adopted in staining after which analysis with an enhanced chemiluminescence light detecting kit (Lianke Multi Sciences, Hangzhou, China). GAPDH was used as control. Signal intensity was quantified using ImageJ software [37,38].

### 4.7. Lipid Staining and Intracellular Triglyceride Quantification

Cellular lipid staining was performed as previously reported [39]. After certain treatment, cells were fixed in 4% formaldehyde for 10 min and then wash three times with phosphate-buffered saline (PBS). After washing, cells were incubated with Oil Red O staining solution (in 60% isopropanol) for 10 min and washed with 60% isopropanol once and with then PBS twice, hematoxylin was added for nuclear staining, after which three timesPBS washing was performed, cells were inspected under light microscopy (Nikon NTY-MV-4000 A, Kyoto, Japan) at 40×.

Intracellular TG was quantified as previously described [39]. Cells were collected for intracellular TG determination using a commercial kit (Applygen, Beijing, China), according to the manufacturer’s instructions. Cellular TG content was normalized by total protein.

### 4.8. Bio-Informatic Analysis of LncRNA Targets and Associated Pathways

Previously reported algorithms of predicting *cis*/*trans*-regulatory effects were used to identify target genes of lncRNA [40]. LncRNA and potential target genes were paired and visualized using UCSC genome browser [41]. The genes transcribed within a 10 kbp window upstream or downstream of lncRNAs location were considered as *cis*-target genes [42]. For *trans*-target gene analyses, RNAplex v0.2, which is a fast tool for RNA–RNA interaction searches by neglecting intramolecular interactions and using lightly simplified energy model, was used [43]. RNAplex parameters were set as—e < −20 in current study to identify the trans-associated genes [44], and genes that were found to be located on that same chromosome as the lncRNA were excluded [45].

Gene Ontology analysis were conducted with GeneCodis web tool [46] and permutated *p*-value cut-off was set as below 0.05 [47]. Pathway enrichment analysis of mRNAs and lncRNA targets was conducted through Integrated Discovery v6.7 functional annotation clustering [48].

### 4.9. Statistical Analysis

Average fold change of four samples were used. Low intensity filters were set at five for lncRNA and six for mRNA to remove less reliable data with Microsoft Office Excel 2013 (Microsoft, Redmond, WA, USA). Statistical significance of differences was determined using Student’s *t*-test. Statistical analysis was performed using SPSS 20 (IBM, Chicago, IL, USA). The significance level was set at 0.05 in the current study and *p*-values less than 0.05 were considered significant, unless otherwise stated.

## 5. Conclusions

In this study, we characterized global changes in lncRNAs and mRNA in an NAFLD mice model. Eighty-nine upregulated and 177 downregulated mRNAs were identified, together with 291 deregulated lncRNAs, consisting of 111 increased and 180 decreased lncRNAs. The function of these changes has not been fully elucidated, but the potential pathways involved were predicted, such as arachidonic acid metabolism, circadian rhythm, linoleic acid metabolism, PPAR signaling pathway, sphingolipid metabolism, steroid biosynthesis, tryptophan metabolism, and tyrosine metabolism. Interference of the lncRNA FLRL2 expression affected the predicted target, circadian rhythm *Arntl* gene expression level, indicating a new aspect of NAFLD. Further investigations are still needed concerning lncRNA in NAFLD. First, we will dedicate our efforts to investigating how FLRL2 interacts with the *Arntl* gene by RNA immunoprecipitation (RIP)/chromatin isolation by RNA purification (ChIRP) in order to better understand the mechanism of FLRL2 in NAFLD. Second, we will explore other lncRNA functions and mechanisms in NAFLD, such as FLRL6, FLRL5, etc., to unveil the whole new lncRNA in NAFLD. Third, with a relatively clear picture of FLRLs in NAFLD pathogenesis, we hope to apply lncRNA in a clinical setting, such as constructing a lncRNA-based non-invasive system for NAFLD diagnosis, developing an lncRNA-based prognosis predicting system, or exploiting new therapeutic small molecules targeting lncRNA in NAFLD treatment. The study we have presented here is only a preliminary, and we believe the role of lncRNA in NAFLD will gradually become more vivid.

## Supplementary Materials

Supplementary materials can be found at www.mdpi.com/1422-0067/18/1/21/s1.
