# Supplementary material for: Long Non-Coding RNA Profiling in a Non-Alcoholic Fatty Liver Disease Rodent Model: New Insight into Pathogenesis
Source: Int J Mol Sci. 2017 Jan 16;18(1):21. doi: 10.3390/ijms18010021 (PMC5297656; doi:10.3390/ijms18010021)
Supplement: Supplementary file 1 [file ijms-18-00021-s001.zip › ijms-155782-Supplementary Materials/ijms-155782_final_supplementary.pdf]

Supplementary Materials: Long Non-Coding RNA Profiling in a Non-Alcoholic Fatty Liver Disease Rodent Model: New Insight into Pathogenesis

Yi Chen, Haixiu Huang, Chengfu Xu, Chaohui Yu and Youming Li

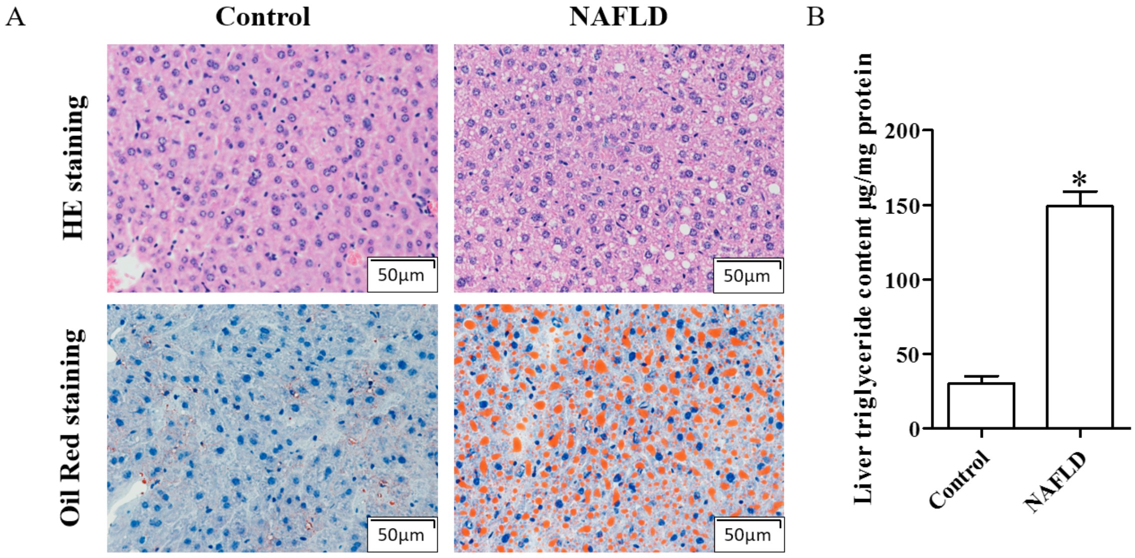

Figure S1. Pathological changes of an NAFLD rodent model. (**A**) Hematoxylin–eosin (HE) staining and Oil Red staining of NAFLD mice liver. Scale bar: 50 μm; (**B**) Liver triglyceride content of NAFLD and control mice. Data are presented as the mean ± SD. * $p < 0.001$.

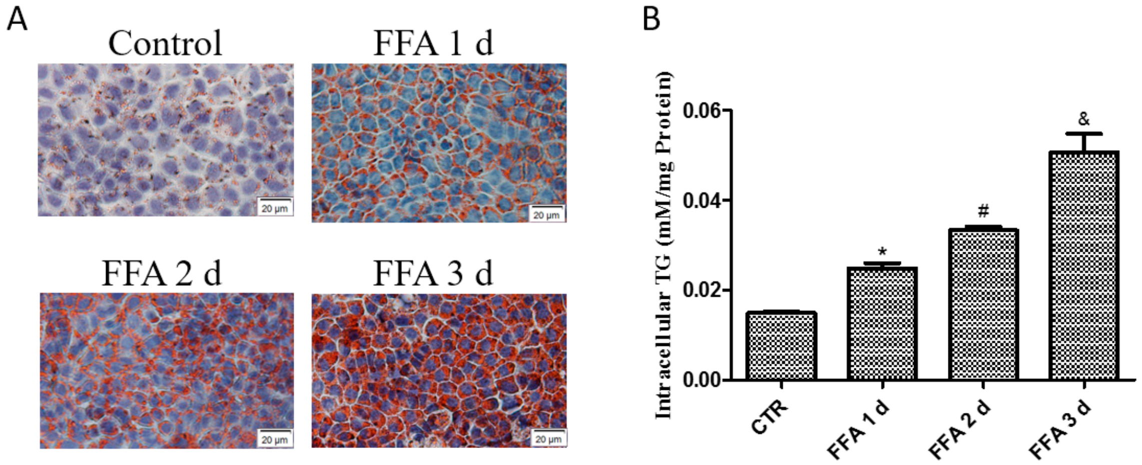

Figure S2. Pathological changes of an NAFLD cellular model. (**A**) Oil Red staining of FFA-treated AML12 cells, at 1 day (d), 2 days, 3 days. Scale bar: 20 μm; (**B**) Intracellular TG content of NAFLD cells and control.Data are presented as the mean ± SD. * $p < 0.001$, compared with control group; # $p < 0.001$, compared with FFA 1d group; & $p < 0.001$, compared with the FFA 2d group.

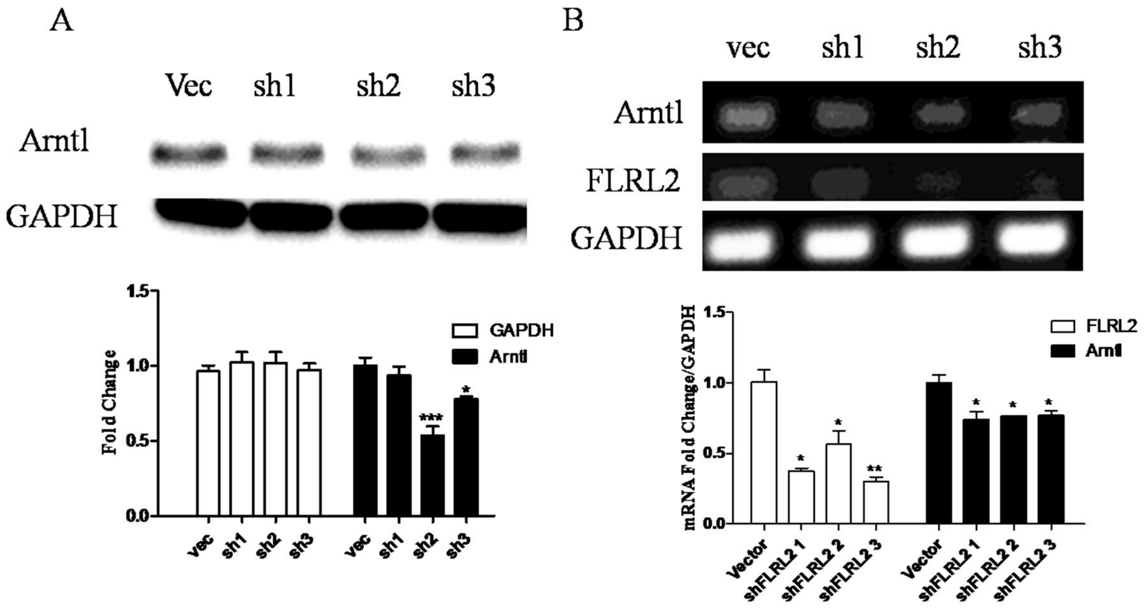

Figure S3. Knockdown of FLRL2 downregulated predicted target *Arntl* expression. Three shFLRL2 plasmids (sh1–3) or an empty vector as a control were tranfected in AML12 cells for 48 h. Cell extracts were prepared for Western blot (**A**) and qPCR (**B**). mRNA levels of FLRL2 and Arntl were measured by qPCR, normalized to GAPDH and presented as the mean ± SD relative to the levels of control cells from three experiments. * $p < 0.05$, ** $p < 0.01$, *** $p < 0.001$.

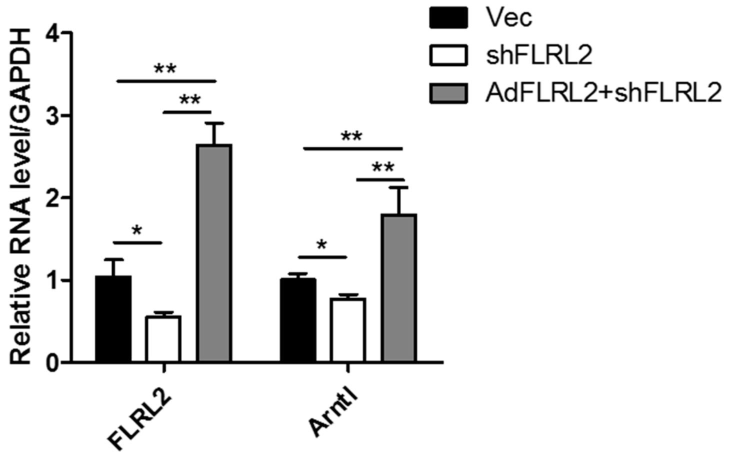

Figure S4. Rescue experiment of *FLRL2* knockdown. The plasmids shFLRL2 and (modified) AdFLRL2, or an empty vector as a control were tranfected in AML12 cells for 48 h. Cell extracts were prepared for qPCR. mRNA levels of FLRL2 and Arntl were measured by qPCR and presented as the mean ± SD relative to the levels of control cells from three experiments, normalized to GAPDH. * $p < 0.05$, ** $p < 0.01$.

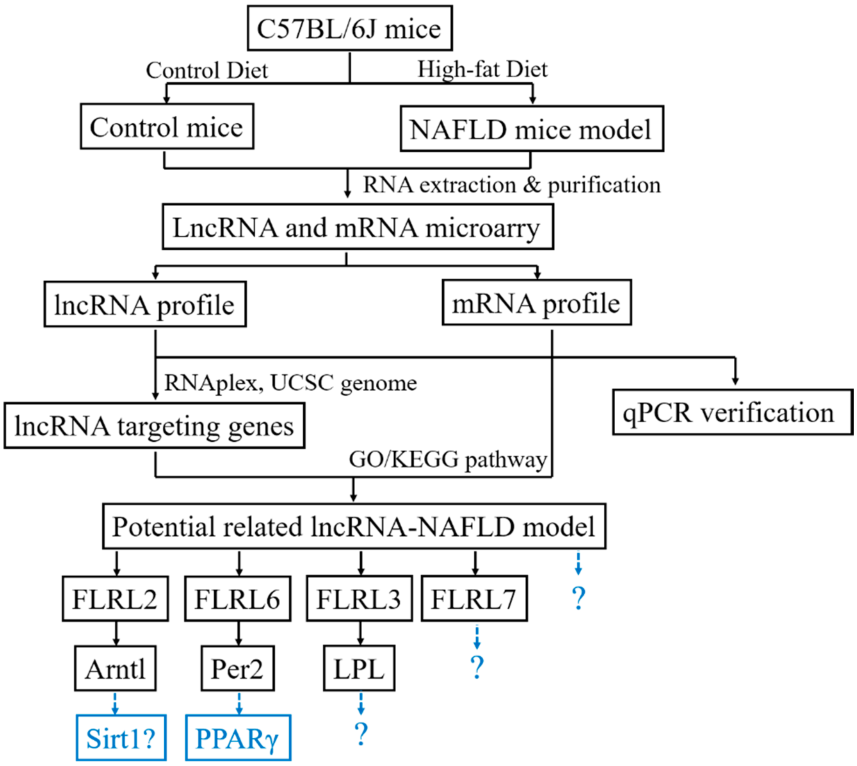

Figure S5. Flowchart of the experimental design.

Table S1. Information of deregulated lncRNAs by microarray analysis.

Probe Name	*p*-Value	Fold Change	Sequence Name	LncRNA Source	LncRNA Acession No-	LncRNA Location
CUST_16222_PI428753551	0.004	11.00	seq5729-3	Fantom3.0	ri∣5330405H16∣PX00053O21∣3487	chr4: 151033075-151036561; −
CUST_20609_PI428753551	0.012	8.46	seq7258-1	Fantom3.0	ri∣6430539F13∣PX00648F05∣1615	Unknown
CUST_85433_PI428753551	0.000	7.74	seq29857-3	UCSC	uc007eim.1	chr10: 7780267-7781356; +
CUST_50555_PI428753551	0.019	6.79	seq17750-1	Fantom3.0	ri∣C130011B08∣PX00666E11∣1381	chr4: 151042408-151043788; −
CUST_50556_PI428753551	0.011	6.11	seq17750-2	Fantom3.0	ri∣C130011B08∣PX00666E11∣1381	chr4: 151042408-151043788; −
CUST_8232_PI428753551	0.012	5.63	seq2913-3	Fantom3.0	ri∣2810049O06∣ZX00065N17∣968	chr4: 151003656- 151004624; −
CUST_84983_PI428753551	0.012	5.06	seq29706-1	UCSC	uc011wph.1	chr1: 91415981-91459328; −
CUST_45020_PI428753551	0.022	4.66	seq15816-1	Fantom3.0	ri∣B020030O10∣PX00326D23∣1416	Unknown
CUST_85431_PI428753551	0.001	4.41	seq29857-1	UCSC	uc007eim.1	chr10: 7780267-7781356; +
CUST_49093_PI428753551	0.008	4.36	seq17238-2	Fantom3.0	ri∣B930060P07∣PX00164N22∣2100	Unknown
A_30_P01024486	0.045	4.10	chr3:58118325-58136550_R	Aglient G3 8 × 60 K	Unknown	chr3: 58118325-58136550; −
CUST_8230_PI428753551	0.020	3.95	seq2913-1	Fantom3.0	ri∣2810049O06∣ZX00065N17∣968	chr4: 151003656-151004624; −
CUST_20611_PI428753551	0.016	3.94	seq7258-3	Fantom3.0	ri∣6430539F13∣PX00648F05∣1615	Unknown
CUST_54276_PI428753551	0.029	3.87	seq19027-3	Fantom3.0	ri∣C230071P09∣PX00176O09∣1184	chr1: 51148308-51149493; +
CUST_8231_PI428753551	0.024	3.78	seq2913-2	Fantom3.0	ri∣2810049O06∣ZX00065N17∣968	chr4: 151003656-151004624; −
CUST_84984_PI428753551	0.018	3.67	seq29706-2	UCSC	uc011wph.1	chr1: 91415981-91459328; −
CUST_6122_PI428753551	0.013	3.60	seq2165-3	Fantom3.0	ri∣1810044K17∣ZX00081G24∣538	chr16: 93368238-93369029; +
CUST_86955_PI428753551	0.003	3.55	seq30374-2	UCSC	uc007oqy.1	chr12: 98523843-98526583; +
CUST_87368_PI428753551	0.012	3.53	seq30516-2	UCSC	uc007rhj.2	chr13: 80883421-80896043; +
CUST_89336_PI428753551	0.001	3.47	seq31188-1	UCSC	uc008esv.1	chr18: 39362582-39367054; +
CUST_17402_PI428753551	0.011	3.46	seq6139-3	Fantom3.0	ri∣5730583K17∣PX00645M13∣1507	Unknown
CUST_44014_PI428753551	0.001	3.35	seq15454-1	Fantom3.0	ri∣A830098B18∣PX00157A12∣2035	Unknown
A_30_P01032515	0.005	3.32	chr13:67211950-67217199_R	Aglient G3 8 × 60 K	Unknown	chr13: 67211950-67217199; −
CUST_84985_PI428753551	0.014	3.31	seq29706-3	UCSC	uc011wph.1	chr1:91415981-91459328; −
CUST_87369_PI428753551	0.018	3.30	seq30516-3	UCSC	uc007rhj.2	chr13: 80883421-80896043; +
CUST_86956_PI428753551	0.001	3.28	seq30374-3	UCSC	uc007oqy.1	chr12: 98523843-98526583; +
CUST_25101_PI428753551	0.002	3.25	seq8848-1	Fantom3.0	ri∣9130221J18∣PX00061D13∣2012	Unknown
CUST_34061_PI428753551	0.009	3.23	seq11944-3	Fantom3.0	ri∣A230042E21∣PX00692L23∣1370	Unknown
CUST_28754_PI428753551	0.005	3.12	seq10115-3	Fantom3.0	ri∣9530055D10∣PX00653H14∣2290	chr6: 145857293-145859583; −
CUST_49094_PI428753551	0.004	3.11	seq17238-3	Fantom3.0	ri∣B930060P07∣PX00164N22∣2100	Unknown
CUST_62847_PI428753551	0.001	2.93	seq21992-1	Fantom3.0	ri∣D630008D04∣PX00196M03∣1007	Unknown
CUST_25103_PI428753551	0.002	2.91	seq8848-3	Fantom3.0	ri∣9130221J18∣PX00061D13∣2012	Unknown
CUST_63021_PI428753551	0.042	2.85	seq22053-3	Fantom3.0	ri∣D630020N23∣PX00197O18∣1929	chr19: 18638350-18640279; +
CUST_88089_PI428753551	0.003	2.81	seq30763-3	UCSC	uc007wgq.1	chr15: 75131376-75133807; −
CUST_52740_PI428753551	0.025	2.75	seq18500-3	Fantom3.0	ri∣C130087E15∣PX00172D19∣2060	Unknown

Table S1. *Cont.*

Probe Name	*p*-Value	Fold Change	Sequence Name	LncRNA Source	LncRNA Acession No-	LncRNA Location
CUST_16221_PI428753551	0.019	2.71	seq5729-2	Fantom3.0	ri|5330405H16|PX00053O21|3487	chr4: 151033075-151036561; −
CUST_6974_PI428753551	0.008	2.68	seq2469-3	Fantom3.0	ri|2310028D20|ZX00039H19|1738	chr15: 81825121-81826859; =
CUST_31121_PI428753551	0.036	2.66	seq10920-3	Fantom3.0	ri|A030006L04|PX00063O10|3044	chr4:32285689-32288735; +
CUST_56589_PI428753551	0.000	2.64	seq19828-3	Fantom3.0	ri|C730038H21|PX00740C01|2784	Unknown
CUST_65266_PI428753551	0.013	2.64	seq22837-2	Fantom3.0	ri|D930024J08|PX00202F21|1435	chr15: 22882397-22883831; +
CUST_16264_PI428753551	0.015	2.62	seq5744-3	Fantom3.0	ri|5330409N07|PX00053M17|2806	Unknown
CUST_59265_PI428753551	0.017	2.60	seq20748-2	Fantom3.0	ri|D130070L05|PX00186E11|2248	Unknown
CUST_37986_PI428753551	0.000	2.59	seq13340-3	Fantom3.0	ri|A530016B22|PX00140L19|752	Unknown
CUST_69671_PI428753551	0.020	2.58	seq24368-1	Fantom3.0	ri|E330039K20|PX00319A15|2183	chr9: 46239840-46242032; −
CUST_48087_PI428753551	0.033	2.57	seq16888-3	Fantom3.0	ri|B930001F21|PX00162H09|2944	Unknown
CUST_1019_PI428753551	0.023	2.57	seq362-2	Ensembl	ENSMUST00000143291	chr11: 119220160-119222473; +
CUST_6509_PI428753551	0.001	2.53	seq2304-1	Fantom3.0	ri|2200003J05|R000023F20|593	chr8: 61395402-61395993; −
CUST_15820_PI428753551	0.006	2.53	seq5589-2	Fantom3.0	ri|4933433F08|PX00021J17|1171	Unknown
CUST_6973_PI428753551	0.009	2.49	seq2469-2	Fantom3.0	ri|2310028D20|ZX00039H19|1738	chr15: 81825121-81826859; +
CUST_46057_PI428753551	0.008	2.48	seq16184-3	Fantom3.0	ri|B230206C11|PX00718B14|2474	Unknown
CUST_73659_PI428753551	0.001	2.47	seq25789-3	Fantom3.0	ri|F830104F12|PM00025A21|2735	chr2: 155826144-155828880; +
CUST_91614_PI428753551	0.001	2.44	seq31960-3	UCSC	uc008vex.2	chr4: 134356372-134372547; −
CUST_25102_PI428753551	0.007	2.43	seq8848-2	Fantom3.0	ri|9130221J18|PX00061D13|2012	Unknown
CUST_88755_PI428753551	0.015	2.38	seq30990-1	UCSC	uc008afl.2	chr17: 5941279-6038555; +
CUST_68779_PI428753551	0.016	2.37	seq24055-3	Fantom3.0	ri|E230024E19|PX00210C21|2564	Unknown
CUST_88757_PI428753551	0.012	2.35	seq30990-3	UCSC	uc008afl.2	chr17: 5941279-6038555; +
CUST_3494_PI428753551	0.001	2.35	seq1229-3	Fantom3.0	ri|1110020M10|R000016P19|1047	Unknown
CUST_35718_PI428753551	0.038	2.35	seq12532-1	Fantom3.0	ri|A330065D06|PX00132H01|2720	chr7: 110122743-110143283; +
CUST_3069_PI428753551	0.012	2.34	seq1083-3	Fantom3.0	ri|0610039G09|R000004L11|597	Unknown
CUST_56299_PI428753551	0.035	2.34	seq19727-1	Fantom3.0	ri|C730004J18|PX00698C15|2764	Unknown
CUST_63019_PI428753551	0.036	2.34	seq22053-1	Fantom3.0	ri|D630020N23|PX00197O18|1929	chr19: 18638350-18640279; +
CUST_48179_PI428753551	0.011	2.33	seq16919-3	Fantom3.0	ri|B930006H01|PX00162O08|2572	Unknown
CUST_94177_PI428753551	0.001	2.32	seq32828-3	UCSC	uc012ggw.1	chr8: 84001705-84004770; +
CUST_53390_PI428753551	0.021	2.30	seq18725-1	Fantom3.0	ri|C230024E18|PX00173L17|3174	Unknown
CUST_48086_PI428753551	0.015	2.28	seq16888-2	Fantom3.0	ri|B930001F21|PX00162H09|2944	Unknown
CUST_24565_PI428753551	0.023	2.28	seq8662-3	Fantom3.0	ri|8430438G05|PX00714L21|1416	Unknown
CUST_89337_PI428753551	0.002	2.28	seq31188-2	UCSC	uc008esv.1	chr18: 39362582-39367054; +
CUST_46055_PI428753551	0.037	2.28	seq16184-1	Fantom3.0	ri|B230206C11|PX00718B14|2474	Unknown
CUST_37985_PI428753551	0.000	2.27	seq13340-2	Fantom3.0	ri|A530016B22|PX00140L19|752	Unknown
CUST_79983_PI428753551	0.021	2.27	seq28010-3	NCBI Refseq	gi|339515665|ref|NR_040310.1|	Unknown

Table S1. *Cont.*

Probe Name	*p*-Value	Fold Change	Sequence Name	LncRNA Source	LncRNA Acession No-	LncRNA Location
CUST_80640_PI428753551	0.042	2.25	seq28235-2	NCBI Refseq	gi∣325995141∣ref∣NR_037956.1∣	Unknown
CUST_38958_PI428753551	0.015	2.25	seq13682-1	Fantom3.0	ri∣A530087K12∣PX00143G22∣2336	chr2: 57368383-57370719; +
CUST_88567_PI428753551	0.013	2.24	seq30925-3	UCSC	uc007zbn.1	chr16: 35658439-35662898; +
CUST_92788_PI428753551	0.010	2.24	seq32359-1	UCSC	uc009cvm.1	chr6: 88716423-88725860; −
CUST_92790_PI428753551	0.003	2.23	seq32359-3	UCSC	uc009cvm.1	chr6: 88716423-88725860; −
CUST_73255_PI428753551	0.030	2.23	seq25644-1	Fantom3.0	ri∣F830034C20∣PS00004O21∣1390	chr15: 7199925-7201309; +
CUST_88933_PI428753551	0.000	2.23	seq31050-3	UCSC	uc008bun.1	chr17: 31152751-31163052; +
CUST_15854_PI428753551	0.023	2.23	seq5601-1	Fantom3.0	ri∣4933433O05∣PX00642I22∣2085	Unknown
CUST_6972_PI428753551	0.017	2.23	seq2469-1	Fantom3.0	ri∣2310028D20∣ZX00039H19∣1738	chr15: 81825121-81826859; +
CUST_53392_PI428753551	0.013	2.23	seq18725-3	Fantom3.0	ri∣C230024E18∣PX00173L17∣3174	Unknown
CUST_6236_PI428753551	0.010	2.22	seq2205-3	Fantom3.0	ri∣2010002C16∣ZX00057O18∣689	Unknown
A_30_P01026616	0.029	2.22	chr7:134649844-134651551_F	Aglient G3 8 × 60 K	Unknown	chr7: 134649844-134651551; +
A_30_P01031796	0.001	2.21	chr6:35201798-35217831_R	Aglient G3 8 × 60 K	Unknown	chr6: 35201798-35217831; −
CUST_46056_PI428753551	0.002	2.19	seq16184-2	Fantom3.0	ri∣B230206C11∣PX00718B14∣2474	Unknown
A_30_P01020529	0.044	2.18	chr1:64686823-64728598_F	Aglient G3 8 × 60 K	Unknown	chr1: 64686823-64728598; +
CUST_84717_PI428753551	0.027	2.18	seq29616-1	UCSC	uc007aet.1	chr1: 3205903-3215632; −
CUST_91879_PI428753551	0.045	2.16	seq32049-3	UCSC	uc008wti.2	chr5: 26460069-26493314; −
CUST_85432_PI428753551	0.003	2.14	seq29857-2	UCSC	uc007eim.1	chr10: 7780267-7781356; +
CUST_56656_PI428753551	0.026	2.13	seq19851-2	Fantom3.0	ri∣C730047E03∣PX00087F06∣2840	Unknown
CUST_52687_PI428753551	0.013	2.12	seq18482-3	Fantom3.0	ri∣C130085F16∣PX00172M20∣3120	Unknown
CUST_89631_PI428753551	0.035	2.12	seq31288-2	UCSC	uc008gpa.1	chr19:10063631-10064017; −
CUST_37984_PI428753551	0.001	2.12	seq13340-1	Fantom3.0	ri∣A530016B22∣PX00140L19∣752	Unknown
CUST_39807_PI428753551	0.008	2.12	seq13981-3	Fantom3.0	ri∣A630035A22∣PX00145E21∣2301	Unknown
CUST_20043_PI428753551	0.028	2.11	seq7059-3	Fantom3.0	ri∣6330570G11∣PX00717G08∣2177	Unknown
CUST_89632_PI428753551	0.042	2.10	seq31288-3	UCSC	uc008gpa.1	chr19: 10063631-10064017; −
CUST_89630_PI428753551	0.032	2.09	seq31288-1	UCSC	uc008gpa.1	chr19: 10063631-10064017; −
CUST_92275_PI428753551	0.036	2.08	seq32184-2	UCSC	uc008znd.1	chr5: 123103043-123132629; −
CUST_87267_PI428753551	0.046	2.07	seq30481-1	UCSC	uc007qsa.1	chr13: 55775946-55784479; −
CUST_19210_PI428753551	0.035	2.06	seq6769-2	Fantom3.0	ri∣6030497D23∣PX00058B20∣1541	Unknown
CUST_78296_PI428753551	0.036	2.05	seq27440-1	NCBI Refseq	gi∣344313180∣ref∣NR_045032.1∣	Unknown
CUST_3428_PI428753551	0.047	2.05	seq1206-2	Fantom3.0	ri∣1110018D14∣R000016D15∣339	chr2: 164564180-164568494; +
CUST_84323_PI428753551	0.001	2.04	seq29484-1	ucr	uc.414-	chr11: 98768057-98768303; −
CUST_48995_PI428753551	0.045	2.04	seq17205-3	Fantom3.0	ri∣B930054E24∣PX00164L12∣2279	chr15: 59076743-59079022; −
CUST_50489_PI428753551	0.042	2.04	seq17727-2	Fantom3.0	ri∣C130006C19∣PX00167J04∣2090	Unknown
CUST_88932_PI428753551	0.000	2.03	seq31050-2	UCSC	uc008bun.1	chr17: 31152751-31163052; +

Table S1. *Cont.*

Probe Name	*p*-Value	Fold Change	Sequence Name	LncRNA Source	LncRNA Acession No-	LncRNA Location
CUST_34060_PI428753551	0.001	2.02	seq11944-1	Fantom3.0	ri|A230042E21|PX00692L23|1370	Unknown
CUST_54584_PI428753551	0.024	2.02	seq19135-2	Fantom3.0	ri|C230084J24|PX00177I12|2679	Unknown
CUST_8303_PI428753551	0.003	2.02	seq2937-2	Fantom3.0	ri|2810408I11|ZX00046B17|713	chr1: 64679870-64690659; −
CUST_15735_PI428753551	0.006	2.01	seq5560-3	Fantom3.0	ri|4933429D07|PX00021K03|2483	chr15: 85803106-85805588; +
CUST_41286_PI428753551	0.044	2.01	seq14493-1	Fantom3.0	ri|A730014F12|PX00149E03|2760	Unknown
CUST_32132_PI428753551	0.032	2.00	seq11271-3	Fantom3.0	ri|A130035K06|PX00122H17|1738	Unknown
CUST_11040_PI428753551	0.002	0.50	seq3890-3	Fantom3.0	ri|4833421B09|PX00028G19|1261	Unknown
CUST_81754_PI428753551	0.028	0.50	seq28613-3	NCBI Refseq	gi|407971021|ref|NR_073190.1|	Unknown
CUST_40335_PI428753551	0.018	0.50	seq14165-2	Fantom3.0	ri|A630059D12|PX00146J13|2639	Unknown
CUST_90586_PI428753551	0.000	0.49	seq31611-2	UCSC	uc008nqe.1	chr2: 158375637-158386145; +
CUST_1594_PI428753551	0.019	0.49	seq563-3	Ensembl	ENSMUST00000136322	chr9: 44478778-44482180; +
CUST_40336_PI428753551	0.005	0.49	seq14165-3	Fantom3.0	ri|A630059D12|PX00146J13|2639	Unknown
CUST_7927_PI428753551	0.037	0.49	seq2804-2	Fantom3.0	ri|2700004G04|ZX00062J14|315	chr1: 167371968-167372282; −
A_30_P01032333	0.017	0.49	chr8:10886200-10905925_F	Aglient G3 8 × 60 K	Unknown	chr8: 10886200-10905925; +
CUST_88347_PI428753551	0.032	0.49	seq30851-3	UCSC	uc011zzj.1	chr15: 100353199-100384435; +
CUST_94429_PI428753551	0.013	0.49	seq32915-1	UCSC	uc029wwz.1	chr8: 129263640-129271868; +
CUST_68590_PI428753551	0.002	0.49	seq23987-2	Fantom3.0	ri|E230014A17|PX00675N03|3275	Unknown
CUST_75433_PI428753551	0.035	0.48	seq26422-3	Fantom3.0	ri|G430129M11|PH00002I21|1112	Unknown
CUST_94570_PI428753551	0.020	0.48	seq32963-3	UCSC	uc009pfk.1	chr9: 45182014-45192968; +
CUST_50209_PI428753551	0.006	0.48	seq17630-2	Fantom3.0	ri|C030033M12|PX00074J01|1267	chr7: 97691503-97692767; +
CUST_69888_PI428753551	0.011	0.48	seq24446-3	Fantom3.0	ri|E430014L16|PX00098F03|3497	Unknown
CUST_68615_PI428753551	0.023	0.48	seq23996-2	Fantom3.0	ri|E230014O04|PX00209J17|2757	Unknown
CUST_6890_PI428753551	0.000	0.48	seq2441-1	Fantom3.0	ri|2310015A16|ZX00039C04|775	chr17: 31907878-31909029; −
A_30_P01029699	0.013	0.48	chr12:77544397-77564122_F	Aglient G3 8 × 60 K	Unknown	chr12: 77544397-77564122; +
CUST_1570_PI428753551	0.022	0.48	seq555-3	Ensembl	ENSMUST00000159268	chr9: 14845613-14847835; +
A_30_P01029722	0.002	0.48	chr13:41337973-41379821_R	Aglient G3 8 × 60 K	Unknown	chr13:41337973-41379821; −
CUST_43541_PI428753551	0.006	0.48	seq15286-3	Fantom3.0	ri|A830039N02|PX00661D20|1825	chrX: 36365031-36366856; +
CUST_93501_PI428753551	0.003	0.48	seq32599-2	UCSC	uc009idd.1	chr7: 82646167-82648528; −
CUST_95272_PI428753551	0.001	0.48	seq33201-2	UCSC	uc029xog.1	chrX: 164402330-164402650; +
CUST_31670_PI428753551	0.037	0.48	seq11109-2	Fantom3.0	ri|A130020J19|PX00121N05|1799	Unknown
CUST_8371_PI428753551	0.022	0.48	seq2962-1	Fantom3.0	ri|2810425J22|ZX00066E19|1403	chr5: 118008789-118010193; +
A_30_P01020035	0.025	0.47	chr4:88745770-88774130_R	Aglient G3 8 × 60 K	Unknown	chr4: 88745770-88774130; −
CUST_28863_PI428753551	0.003	0.47	seq10152-2	Fantom3.0	ri|9530065L16|PX00113I24|1053	chr15: 62039256-62249218; +
CUST_56688_PI428753551	0.004	0.47	seq19862-2	Fantom3.0	ri|C730049O14|PX00087L24|1634	Unknown
A_30_P01027654	0.001	0.47	chr15:62046023-62082530_F	Aglient G3 8 × 60 K	Unknown	chr15: 62046023-62082530; +

Table S1. *Cont.*

Probe Name	*p*-Value	Fold Change	Sequence Name	LncRNA Source	LncRNA Acession No-	LncRNA Location
CUST_25597_PI428753551	0.038	0.47	seq9023-3	Fantom3.0	ri|9230112I12|PX00062C09|1335	chr16: 90440654-90441987; +
CUST_80219_PI428753551	0.001	0.47	seq28090-3	NCBI Refseq	gi|242332555|ref|NR_027961.1|	Unknown
CUST_11277_PI428753551	0.008	0.46	seq3973-3	Fantom3.0	ri|4833445E02|PX00313B08|1768	chr12: 26306800-26308568; −
A_30_P01032931	0.048	0.46	chr12:81217572-81233910_R	Aglient G3 8 × 60 K	Unknown	chr12: 81217572-81233910; −
CUST_86465_PI428753551	0.001	0.46	seq30208-2	UCSC	uc007lgb.1	chr11: 98350861-98357795; +
CUST_93918_PI428753551	0.044	0.46	seq32741-2	UCSC	uc009kux.1	chr8: 10899921-10903874; +
CUST_68220_PI428753551	0.005	0.46	seq23859-3	Fantom3.0	ri|E130217H01|PX00675K21|3578	Unknown
CUST_31671_PI428753551	0.025	0.46	seq11109-3	Fantom3.0	ri|A130020J19|PX00121N05|1799	Unknown
CUST_65027_PI428753551	0.005	0.46	seq22754-3	Fantom3.0	ri|D930018I20|PX00201L09|607	Unknown
CUST_64023_PI428753551	0.006	0.45	seq22407-3	Fantom3.0	ri|D830016D23|PX00198L15|3164	Unknown
CUST_59049_PI428753551	0.003	0.45	seq20675-3	Fantom3.0	ri|D130061L17|PX00185J03|2602	Unknown
CUST_3022_PI428753551	0.000	0.45	seq1067-1	Fantom3.0	ri|0610011H04|R000002H19|401	chr4: 154023310-154025651; 0
CUST_38852_PI428753551	0.001	0.45	seq13644-3	Fantom3.0	ri|A530083F01|PX00143I09|1014	Unknown
CUST_62203_PI428753551	0.006	0.45	seq21763-3	Fantom3.0	ri|D430033D05|PX00195G04|3827	chr4: 63135007-63138829; +
CUST_68591_PI428753551	0.004	0.45	seq23987-3	Fantom3.0	ri|E230014A17|PX00675N03|3275	Unknown
CUST_2877_PI428753551	0.002	0.45	seq1016-1	Ensembl	ENSMUST00000150851	chr12: 109549255-109549775; +
CUST_2935_PI428753551	0.025	0.45	seq1036-3	Ensembl	ENSMUST00000135144	chr3: 89315439-89318907; +
A_30_P01031483	0.000	0.45	chr15:62046023-62082530_F	Aglient G3 8 × 60 K	Unknown	chr15: 62046023-62082530; +
CUST_6087_PI428753551	0.005	0.45	seq2153-2	Fantom3.0	ri|1810034M13|ZX00155P23|722	chr11: 57970243-57973137; −
CUST_11555_PI428753551	0.001	0.45	seq4069-3	Fantom3.0	ri|4921534G14|PX00713F12|992	Unknown
CUST_88041_PI428753551	0.004	0.45	seq30747-2	UCSC	uc007vyl.1	chr15: 62037986-62258637; +
CUST_94841_PI428753551	0.001	0.44	seq33055-2	UCSC	uc029xfu.1	chr9
CUST_35567_PI428753551	0.012	0.44	seq12478-3	Fantom3.0	ri|A330052A13|PX00742H08|1244	Unknown
CUST_70976_PI428753551	0.012	0.44	seq24831-3	Fantom3.0	ri|F630104J01|PL00015H17|3892	Unknown
CUST_89974_PI428753551	0.003	0.44	seq31404-3	UCSC	uc008irs.1	chr2: 25361491-25365626; −
A_30_P01026769	0.003	0.44	chr3:45081952-45092352_F	Aglient G3 8 × 60 K	Unknown	chr3: 45081952-45092352; +
CUST_3001_PI428753551	0.004	0.44	seq1059-3	Fantom3.0	ri|0610007F13|R000001H01|803	chr4: 115090931-115093863; +
CUST_36448_PI428753551	0.012	0.44	seq12792-2	Fantom3.0	ri|A430025P12|PX00724F15|435	Unknown
A_30_P01018666	0.049	0.43	chr3:146629425-146649125_F	Aglient G3 8 × 60 K	Unknown	chr3: 146629425-146649125; +
A_30_P01025687	0.003	0.43	chr15:61984389-62102500_F	Aglient G3 8 × 60 K	Unknown	chr15: 61984389-62102500; +
CUST_81152_PI428753551	0.008	0.43	seq28410-2	NCBI Refseq	gi|353523863|ref|NR_045347.1|	Unknown
CUST_3024_PI428753551	0.001	0.43	seq1067-3	Fantom3.0	ri|0610011H04|R000002H19|401	chr4: 154023310-15402565; −
CUST_94842_PI428753551	0.000	0.43	seq33055-3	UCSC	uc029xfu.1	chr9: 103086109-103088267; +
CUST_36447_PI428753551	0.019	0.42	seq12792-1	Fantom3.0	ri|A430025P12|PX00724F15|435	Unknown
CUST_70722_PI428753551	0.015	0.42	seq24742-2	Fantom3.0	ri|F530212E09|PS00003N05|1281	Unknown

Table S1. *Cont.*

Probe Name	*p*-Value	Fold Change	Sequence Name	LncRNA Source	LncRNA Acession No-	LncRNA Location
CUST_43540_PI428753551	0.024	0.42	seq15286-2	Fantom3.0	ri|A830039N02|PX00661D20|1825	chrX: 36365031-36366856; +
CUST_91723_PI428753551	0.001	0.42	seq31997-1	UCSC	uc008waz.2	chr4: 154020469-154025621; −
CUST_77946_PI428753551	0.001	0.41	seq27321-1	NCBI Refseq	gi|84781715|ref|NR_002700.1|	Unknown
CUST_65289_PI428753551	0.013	0.41	seq22845-2	Fantom3.0	ri|D930025F21|PX00202H11|2836	Unknown
CUST_42051_PI428753551	0.045	0.41	seq14756-3	Fantom3.0	ri|A730053D01|PX00151A14|586	chr10: 93317402-93322725; +
CUST_93919_PI428753551	0.044	0.41	seq32741-3	UCSC	uc009kux.1	chr8: 10899921-10903874; +
CUST_59048_PI428753551	0.015	0.41	seq20675-2	Fantom3.0	ri|D130061L17|PX00185J03|2602	Unknown
A_30_P01021311	0.000	0.41	chr9:78107225-78118850_R	Aglient G3 8 × 60 K	Unknown	chr9:78107225-78118850; −
CUST_1667_PI428753551	0.001	0.41	seq589-3	Ensembl	ENSMUST00000146054	chr4:154023311-154026150; −
CUST_65290_PI428753551	0.001	0.41	seq22845-3	Fantom3.0	ri|D930025F21|PX00202H11|2836	Unknown
CUST_1421_PI428753551	0.008	0.41	seq505-2	Ensembl	ENSMUST00000150171	chr4:129263730-129265010; +
CUST_34193_PI428753551	0.001	0.40	seq11990-2	Fantom3.0	ri|A230053F13|PX00128A20|3998	chr4:154021460-154026152; −
CUST_6216_PI428753551	0.038	0.40	seq2198-3	Fantom3.0	ri|1810074C23|ZX00081O02|389	chr8: 9417924-94180325; +
CUST_69633_PI428753551	0.024	0.40	seq24355-2	Fantom3.0	ri|E330038F23|PX00312H20|2441	Unknown
CUST_73375_PI428753551	0.032	0.40	seq25685-2	Fantom3.0	ri|F830037N24|PL00007C18|2867	Unknown
CUST_6993_PI428753551	0.002	0.39	seq2476-1	Fantom3.0	ri|2310031A18|ZX00039N07|888	chr8: 124740257-124741144; −
CUST_85313_PI428753551	0.006	0.39	seq29816-3	UCSC	uc007dvi.1	chr1: 178937109-178939199; +
CUST_28392_PI428753551	0.046	0.39	seq9992-2	Fantom3.0	ri|9530024C23|PX00111L16|396	chr7: 4149812-4150208; −
CUST_1664_PI428753551	0.000	0.39	seq588-3	Ensembl	ENSMUST00000145527	chr4: 154023311-154024355; −
CUST_77947_PI428753551	0.002	0.39	seq27321-2	NCBI Refseq	gi|84781715|ref|NR_002700.1|	Unknown
A_30_P01030223	0.025	0.39	chr2:119697845-119719245_F	Aglient G3 8 × 60 K	Unknown	chr2: 119697845-119719245; +
CUST_92466_PI428753551	0.006	0.39	seq32249-1	UCSC	uc009aur.2	chr5:151406503-151432119; +
CUST_70723_PI428753551	0.021	0.39	seq24742-3	Fantom3.0	ri|F530212E09|PS00003N05|1281	Unknown
CUST_76617_PI428753551	0.009	0.39	seq26843-3	Fantom3.0	ri|I1C0003N05|FU00001K12|1299	Unknown
CUST_17832_PI428753551	0.001	0.39	seq6291-2	Fantom3.0	ri|5830447G06|PX00645N15|2148	Unknown
CUST_22296_PI428753551	0.005	0.38	seq7847-3	Fantom3.0	ri|7030418N07|PX00650C18|1744	Unknown
CUST_94569_PI428753551	0.002	0.38	seq32963-2	UCSC	uc009pfk.1	chr9: 45182014-45192968; +
CUST_69848_PI428753551	0.013	0.38	seq24432-3	Fantom3.0	ri|E430013E17|PX00098I12|1138	Unknown
CUST_22295_PI428753551	0.002	0.38	seq7847-2	Fantom3.0	ri|7030418N07|PX00650C18|1744	Unknown
A_30_P01023153	0.023	0.38	chr12:74135916-74142780_F	Aglient G3 8 × 60 K	Unknown	chr12: 74135916-74142780; +
CUST_94066_PI428753551	0.001	0.38	seq32791-2	UCSC	uc009lwr.1	chr8: 68880554-68907448; +
CUST_69201_PI428753551	0.041	0.37	seq24206-1	Fantom3.0	ri|E330015J04|PX00675D12|1730	Unknown
CUST_31052_PI428753551	0.045	0.37	seq10897-3	Fantom3.0	ri|9930118G10|PX00718M15|1380	Unknown
CUST_31128_PI428753551	0.016	0.36	seq10923-1	Fantom3.0	ri|A030007K14|PX00063E18|1900	Unknown
CUST_25755_PI428753551	0.000	0.36	seq9080-1	Fantom3.0	ri|9330010C10|PX00324G22|2717	Unknown

Table S1. *Cont.*

Probe Name	*p*-Value	Fold Change	Sequence Name	LncRNA Source	LncRNA Acession No-	LncRNA Location
CUST_56439_PI428753551	0.007	0.36	seq19775-3	Fantom3.0	ri|C730020I24|PX00086L21|432	chr4: 11611139-11615007; −
CUST_39241_PI428753551	0.013	0.36	seq13781-3	Fantom3.0	ri|A630005I04|PX00143J18|3500	chr7: 113240249-113243748; +
CUST_6995_PI428753551	0.003	0.36	seq2476-3	Fantom3.0	ri|2310031A18|ZX00039N07|888	chr8: 124740257-124741144; −
CUST_6994_PI428753551	0.004	0.36	seq2476-2	Fantom3.0	ri|2310031A18|ZX00039N07|888	chr8: 124740257-124741144; −
A_30_P01021437	0.000	0.36	chr17:32035750-32056000_R	Aglient G3 8 × 60 K	Unknown	chr17: 32035750-32056000; −
CUST_87031_PI428753551	0.011	0.36	seq30399-3	UCSC	uc011yum.1	chr12: 110275384-110278068; −
A_30_P01032927	0.006	0.35	chr3:121615836-121616109_F	Aglient G3 8 × 60 K	Unknown	chr3: 121615836-121616109; +
CUST_94069_PI428753551	0.001	0.35	seq32792-2	UCSC	uc029wtn.1	chr8: 68905542-68906931; +
CUST_56438_PI428753551	0.008	0.35	seq19775-2	Fantom3.0	ri|C730020I24|PX00086L21|432	chr4: 11611139-11615007; −
CUST_94070_PI428753551	0.001	0.35	seq32792-3	UCSC	uc029wtn.1	chr8: 68905542-68906931; +
A_30_P01030951	0.016	0.34	chr8:10886200-10905925_F	Aglient G3 8 × 60 K	Unknown	chr8: 10886200-10905925; +
CUST_77800_PI428753551	0.002	0.34	seq27270-3	lncrnadb	MEG9_EU616813.1	Unknown
CUST_22294_PI428753551	0.002	0.34	seq7847-1	Fantom3.0	ri|7030418N07|PX00650C18|1744	Unknown
A_30_P01029662	0.012	0.33	chr8:10886200-10905925_F	Aglient G3 8 × 60 K	Unknown	chr8: 10886200-10905925; +
CUST_56613_PI428753551	0.009	0.33	seq19836-3	Fantom3.0	ri|C730040I22|PX00087D14|736	Unknown
CUST_77948_PI428753551	0.002	0.33	seq27321-3	NCBI Refseq	gi|84781715|ref|NR_002700.1|	Unknown
CUST_42418_PI428753551	0.000	0.33	seq14890-2	Fantom3.0	ri|A730074L22|PX00661C22|2537	chr4: 81744945-81760133; +
CUST_69632_PI428753551	0.022	0.32	seq24355-1	Fantom3.0	ri|E330038F23|PX00312H20|2441	Unknown
CUST_87022_PI428753551	0.000	0.31	seq30396-3	UCSC	uc011ysp.1	chr12: 109603944-109661711; +
CUST_6892_PI428753551	0.000	0.31	seq2441-3	Fantom3.0	ri|2310015A16|ZX00039C04|775	chr17: 31907878-31909029; −
CUST_77847_PI428753551	0.000	0.31	seq27286-3	lncrnadb	Rian_NR_028261.1	Unknown
CUST_69847_PI428753551	0.012	0.31	seq24432-2	Fantom3.0	ri|E430013E17|PX00098I12|1138	Unknown
A_30_P01019807	0.000	0.30	chr17:32035750-32056000_R	Aglient G3 8 × 60 K	Unknown	chr17: 32035750-32056000; −
CUST_12775_PI428753551	0.008	0.30	seq4507-1	Fantom3.0	ri|4930471I20|PX00032G07|630	chr2: 155547040-155556930; −
A_30_P01019750	0.000	0.30	chr17:32044823-32045974_R	Aglient G3 8 × 60 K	Unknown	chr17: 32044823-32045974; −
CUST_87049_PI428753551	0.038	0.29	seq30405-3	UCSC	uc007pgh.1	chr12: 113259933-113265117; −
A_30_P01024995	0.000	0.29	chr17:32044823-32045974_R	Aglient G3 8 × 60 K	Unknown	chr17: 32044823-32045974; −
CUST_50154_PI428753551	0.039	0.29	seq17611-2	Fantom3.0	ri|C030026A16|PX00074B24|1881	Unknown
CUST_50508_PI428753551	0.001	0.29	seq17733-3	Fantom3.0	ri|C130007E11|PX00167D05|1553	chr12: 109747902-109749455; +
A_30_P01023749	0.000	0.28	chr17:32044823-32045974_R	Aglient G3 8 × 60 K	Unknown	chr17: 32044823-32045974; −
A_30_P01019873	0.002	0.28	chr9:78107225-78118850_R	Aglient G3 8 × 60 K	Unknown	chr9: 78107225-78118850; −
CUST_6891_PI428753551	0.000	0.28	seq2441-2	Fantom3.0	ri|2310015A16|ZX00039C04|775	chr17: 31907878-31909029; −
A_30_P01026799	0.001	0.28	chr12:110894095-110899517_F	Aglient G3 8 × 60 K	Unknown	chr12: 110894095-110899517; +
CUST_88365_PI428753551	0.008	0.28	seq30857-3	UCSC	uc007xuv.1	chr15: 102188542-102203163; −
CUST_80514_PI428753551	0.024	0.27	seq28191-3	NCBI Refseq	gi|262263311|ref|NR_030715.1|	Unknown

Table S1. *Cont.*

<table>
<tr><th>Probe Name</th><th>p-Value</th><th>Fold Change</th><th>Sequence Name</th><th>LncRNA Source</th><th>LncRNA Acession No-</th><th>LncRNA Location</th></tr>
<tr><td>CUST_65424_PI428753551</td><td>0.004</td><td>0.27</td><td>seq22891-3</td><td>Fantom3.0</td><td>ri|D930029A11|PX00700N21|1364</td><td>chr12: 109600820-109602181; +</td></tr>
<tr><td>CUST_69634_PI428753551</td><td>0.011</td><td>0.27</td><td>seq24355-3</td><td>Fantom3.0</td><td>ri|E330038F23|PX00312H20|2441</td><td>Unknown</td></tr>
<tr><td>CUST_6575_PI428753551</td><td>0.010</td><td>0.26</td><td>seq2327-2</td><td>Fantom3.0</td><td>ri|2210022F04|ZX00160I19|1074</td><td>chr12: 21269099-21270170; +</td></tr>
<tr><td>CUST_87025_PI428753551</td><td>0.001</td><td>0.26</td><td>seq30397-3</td><td>UCSC</td><td>uc011ytv.1</td><td>chr12: 109730275-109749457; +</td></tr>
<tr><td>CUST_90587_PI428753551</td><td>0.000</td><td>0.26</td><td>seq31611-3</td><td>UCSC</td><td>uc008nqe.1</td><td>chr2: 158375637-158386145; +</td></tr>
<tr><td>CUST_6576_PI428753551</td><td>0.006</td><td>0.26</td><td>seq2327-3</td><td>Fantom3.0</td><td>ri|2210022F04|ZX00160I19|1074</td><td>chr12: 21269099-21270170; +</td></tr>
<tr><td>A_30_P01018142</td><td>0.001</td><td>0.26</td><td>chr5:106982442-107045717_R</td><td>Aglient G3 8 × 60 K</td><td>Unknown</td><td>chr5: 106982442-107045717; −</td></tr>
<tr><td>CUST_87028_PI428753551</td><td>0.000</td><td>0.25</td><td>seq30398-3</td><td>UCSC</td><td>uc011ytw.1</td><td>chr12: 109730275-109749457; +</td></tr>
<tr><td>CUST_49885_PI428753551</td><td>0.030</td><td>0.25</td><td>seq17517-3</td><td>Fantom3.0</td><td>ri|C030005P03|PX00073B14|2155</td><td>Unknown</td></tr>
<tr><td>CUST_56114_PI428753551</td><td>0.041</td><td>0.25</td><td>seq19661-3</td><td>Fantom3.0</td><td>ri|C630025E23|PX00698M17|4652</td><td>Unknown</td></tr>
<tr><td>CUST_85494_PI428753551</td><td>0.016</td><td>0.23</td><td>seq29878-2</td><td>UCSC</td><td>uc011xbo.1</td><td>chr10: 22273473-22371114; +</td></tr>
<tr><td>A_30_P01032469</td><td>0.003</td><td>0.23</td><td>chr12:110842154-110883594_F</td><td>Aglient G3 8 × 60 K</td><td>Unknown</td><td>chr12: 110842154-110883594; +</td></tr>
<tr><td>A_30_P01033149</td><td>0.002</td><td>0.23</td><td>chr9:68349343-68411643_F</td><td>Aglient G3 8 × 60 K</td><td>Unknown</td><td>chr9: 68349343-68411643; +</td></tr>
<tr><td>CUST_80817_PI428753551</td><td>0.001</td><td>0.22</td><td>seq28296-2</td><td>NCBI Refseq</td><td>gi|341572586|ref|NR_040533.1|</td><td>Unknown</td></tr>
<tr><td>CUST_85495_PI428753551</td><td>0.015</td><td>0.22</td><td>seq29878-3</td><td>UCSC</td><td>uc011xbo.1</td><td>chr10: 22273473-22371114; +</td></tr>
<tr><td>CUST_80818_PI428753551</td><td>0.003</td><td>0.21</td><td>seq28296-3</td><td>NCBI Refseq</td><td>gi|341572586|ref|NR_040533.1|</td><td>Unknown</td></tr>
<tr><td>CUST_16246_PI428753551</td><td>0.048</td><td>0.21</td><td>seq5738-3</td><td>Fantom3.0</td><td>ri|5330407B06|PX00053C20|4056</td><td>Unknown</td></tr>
<tr><td>CUST_31051_PI428753551</td><td>0.018</td><td>0.21</td><td>seq10897-2</td><td>Fantom3.0</td><td>ri|9930118G10|PX00718M15|1380</td><td>Unknown</td></tr>
<tr><td>CUST_2879_PI428753551</td><td>0.003</td><td>0.20</td><td>seq1016-3</td><td>Ensembl</td><td>ENSMUST00000150851</td><td>chr12: 109549255-109549775; +</td></tr>
<tr><td>CUST_81859_PI428753551</td><td>0.006</td><td>0.20</td><td>seq28649-1</td><td>NCBI Refseq</td><td>gi|236460413|ref|NR_027652.1|</td><td>Unknown</td></tr>
<tr><td>CUST_57798_PI428753551</td><td>0.008</td><td>0.20</td><td>seq20245-3</td><td>Fantom3.0</td><td>ri|D030062B12|PX00699M13|1634</td><td>chr12: 109596909-109598539; +</td></tr>
<tr><td>CUST_23550_PI428753551</td><td>0.012</td><td>0.19</td><td>seq8304-1</td><td>Fantom3.0</td><td>ri|7420455A22|PX00708L20|1270</td><td>Unknown</td></tr>
<tr><td>CUST_84736_PI428753551</td><td>0.012</td><td>0.19</td><td>seq29622-3</td><td>UCSC</td><td>uc007ahc.1</td><td>chr1: 9968625-10009130; −</td></tr>
<tr><td>CUST_51361_PI428753551</td><td>0.001</td><td>0.17</td><td>seq18026-3</td><td>Fantom3.0</td><td>ri|C130039C10|PX00169A12|3280</td><td>Unknown</td></tr>
<tr><td>CUST_72581_PI428753551</td><td>0.045</td><td>0.17</td><td>seq25394-3</td><td>Fantom3.0</td><td>ri|F830012H14|PL00005I08|2807</td><td>Unknown</td></tr>
<tr><td>CUST_27422_PI428753551</td><td>0.000</td><td>0.17</td><td>seq9655-3</td><td>Fantom3.0</td><td>ri|9430047F21|PX00109O24|3478</td><td>Unknown</td></tr>
<tr><td>CUST_4794_PI428753551</td><td>0.017</td><td>0.16</td><td>seq1688-1</td><td>Fantom3.0</td><td>ri|1700025J06|ZX00047C01|517</td><td>chr2: 3409046-3419041; −</td></tr>
<tr><td>CUST_4796_PI428753551</td><td>0.000</td><td>0.16</td><td>seq1688-3</td><td>Fantom3.0</td><td>ri|1700025J06|ZX00047C01|517</td><td>chr2: 3409046-3419041; −</td></tr>
<tr><td>CUST_81858_PI428753551</td><td>0.011</td><td>0.16</td><td>seq28648-3</td><td>NCBI Refseq</td><td>gi|381214349|ref|NR_046475.1|</td><td>Unknown</td></tr>
<tr><td>CUST_3303_PI428753551</td><td>0.006</td><td>0.16</td><td>seq1164-2</td><td>Fantom3.0</td><td>ri|1110006E14|R000013H08|1292</td><td>chr12: 109595804-109597096; +</td></tr>
<tr><td>CUST_81861_PI428753551</td><td>0.010</td><td>0.15</td><td>seq28649-3</td><td>NCBI Refseq</td><td>gi|236460413|ref|NR_027652.1|</td><td>Unknown</td></tr>
<tr><td>CUST_78459_PI428753551</td><td>0.007</td><td>0.15</td><td>seq27494-3</td><td>NCBI Refseq</td><td>gi|356995903|ref|NR_045514.1|</td><td>Unknown</td></tr>
<tr><td>CUST_2874_PI428753551</td><td>0.002</td><td>0.15</td><td>seq1015-1</td><td>Ensembl</td><td>ENSMUST00000146701</td><td>chr12: 109545398-109571726; +</td></tr>
<tr><td>A_30_P01028123</td><td>0.000</td><td>0.14</td><td>chr9:78107225-78118850_R</td><td>Aglient G3 8 × 60 K</td><td>Unknown</td><td>chr9: 78107225-78118850; −</td></tr>
<tr><td>A_30_P01023273</td><td>0.000</td><td>0.14</td><td>chr9:78107225-78118850_R</td><td>Aglient G3 8 × 60 K</td><td>Unknown</td><td>chr9: 78107225-78118850; −</td></tr>
</table>

Table S1. *Cont.*

Probe Name	*p*-Value	Fold Change	Sequence Name	LncRNA Source	LncRNA Acession No-	LncRNA Location
A_30_P01020822	0.001	0.14	chr12:110909258-110913909_F	Aglient G3 8 × 60 K	Unknown	chr12: 110909258-110913909; +
CUST_77846_PI428753551	0.009	0.14	seq27286-2	lncrnadb	Rian_NR_028261.1	Unknown
A_30_P01020973	0.011	0.14	chr5:106982442-107045717_R	Aglient G3 8 × 60 K	Unknown	chr5: 106982442-107045717; −
CUST_87021_PI428753551	0.013	0.13	seq30396-2	UCSC	uc011ysp.1	chr12: 109603944-109661711; +
CUST_16282_PI428753551	0.002	0.12	seq5750-3	Fantom3.0	ri∣5330411G14∣PX00643E15∣1907	chr12: 109737779-109741503; +
CUST_4795_PI428753551	0.001	0.12	seq1688-2	Fantom3.0	ri∣1700025J06∣ZX00047C01∣517	chr2: 3409046-3419041; −
A_30_P01028766	0.002	0.12	chr9:78107225-78118850_F	Aglient G3 8 × 60 K	Unknown	chr9: 78107225-78118850; +
A_30_P01018914	0.003	0.11	chr9:78107225-78118850_F	Aglient G3 8 × 60 K	Unknown	chr9: 78107225-78118850; +
CUST_72580_PI428753551	0.046	0.11	seq25394-2	Fantom3.0	ri∣F830012H14∣PL00005I08∣2807	Unknown
CUST_67599_PI428753551	0.001	0.10	seq23646-3	Fantom3.0	ri∣E130013L12∣PX00207F14∣4523	Unknown
CUST_85493_PI428753551	0.003	0.07	seq29878-1	UCSC	uc011xbo.1	chr10: 22273473-22371114; +

-, antisense strand; +, sense strand.

Table S4. GO pathway categories of potential *cis*/*trans*-targets from significantly upregulated and downregulated lncRNAs in an NAFLD animal model.

Category	GO_Term	*p*-Value
***Cis*-targets of upregulated lncRNAs:**		
Biological Process	Regulation of transcription from RNA polymerase II promoter	7.40×10^{-4}
	Rhythmic process	0.006526
	Fatty acid metabolic process	0.021704
	Negative regulation of specific transcription from RNA polymerase II promoter	0.025966
	Negative regulation of gene-specific transcription	0.028406
	Regulation of transcription, DNA-dependent	0.029616
	Regulation of RNA metabolic process	0.03145
	Negative regulation of transcription from RNA polymerase II promoter	0.03309
	Circadian rhythm	0.045322
Molecular Function	Oxidoreductase activity	0.010788

Table S4. *Cont.*

Category	GO_Term	*p*-Value
***Trans*-targets of upregulated lncRNAs:**		
Biological Process	Translation	7.02×10^{-4}
	Carboxylic acid transport	0.002599
	Organic acid transport	0.002767
	Transcription	0.005228
	Response to ionizing radiation	0.005671
	Antigen processing and presentation of peptide antigen via MHC class I	0.007248
	Apoptosis	0.008696
	Antigen processing and presentation of peptide antigen	0.009478
	Programmed cell death	0.010298
	Cell death	0.010779
	Regulation of transcription	0.013117
	Death	0.013817
	Chromosome segregation	0.018543
	M phase of mitotic cell cycle	0.018583
	Response to radiation	0.021978
	Regulation of JNK cascade	0.022394
	Regulation of stress-activated protein kinase signaling pathway	0.024077
	Positive regulation of protein kinase activity	0.029813
	Mitotic cell cycle	0.033296
	Regulation of cellular response to stress	0.034011
	Positive regulation of kinase activity	0.037137
	Vesicle organization	0.037478
	Response to abiotic stimulus	0.039562
	M phase	0.040202
	DNA repair	0.040945
	Response to DNA damage stimulus	0.043932
	Positive regulation of transferase activity	0.044075
	Transmission of nerve impulse	0.045742

Table S4. *Cont.*

Category	GO_Term	*p*-Value
Cellular Component	Non-membrane-bounded organelle	4.56×10^{-4}
	Intracellular non-membrane-bounded organelle	4.56×10^{-4}
	Condensed chromosome, centromeric region	5.54×10^{-4}
	Nucleolus	0.001334
	Chromosome, centromeric region	0.001389
	Intracellular organelle lumen	0.002934
	Organelle lumen	0.003094
	Kinetochore	0.003613
	Ribonucleoprotein complex	0.003792
	Membrane-enclosed lumen	0.005536
	Mitochondrial lumen	0.00595
	Mitochondrial matrix	0.00595
	Ribosome	0.006654
	Condensed chromosome kinetochore	0.008615
	Large ribosomal subunit	0.013807
	Condensed chromosome	0.014472
	Outer kinetochore of condensed chromosome	0.0146
	Mitochondrion	0.015452
	Mitochondrial ribosome	0.016379
	Organellar ribosome	0.016379
	Organellar large ribosomal subunit	0.016974
	Mitochondrial large ribosomal subunit	0.016974

Table S4. *Cont.*

Category	GO_Term	*p*-Value
Molecular Function	Transition metal ion binding	2.29×10^{-6}
	Cation binding	1.26×10^{-5}
	Metal ion binding	1.35×10^{-5}
	Ion binding	1.42×10^{-5}
	Zinc ion binding	1.88×10^{-4}
	Structural constituent of ribosome	0.002423
	Cytokine receptor activity	0.008179
	Cytokine binding	0.01522
	Lanosterol *O*-acyltransferase activity	0.045087
	Ergosterol *O*-acyltransferase activity	0.045087
	Ribosylnicotinamide kinase activity	0.045087
	Sterol *O*-acyltransferase activity	0.045087
	Cholesterol *O*-acyltransferase activity	0.045087
***Cis*-targets of downregulated lncRNAs:**		
Biological Process	Regulation of transcription from RNA polymerase II promoter	7.40×10^{-4}
	Rhythmic process	0.006526
	Fatty acid metabolic process	0.021704
	Negative regulation of specific transcription from RNA polymerase II promoter	0.025966
	Negative regulation of gene-specific transcription	0.028406
	Regulation of transcription, DNA-dependent	0.029616
	Regulation of RNA metabolic process	0.03145
	Negative regulation of transcription from RNA polymerase II promoter	0.03309
	Circadian rhythm	0.045322
Molecular Function	oxidoreductase activity	0.010788

Table S4. *Cont.*

Category	GO_Term	*p*-Value
***Trans*-targets of downregulated lncRNAs:**		
Biological Process	Translation	7.02×10^{-4}
	Carboxylic acid transport	0.002599
	Organic acid transport	0.002767
	Transcription	0.005228
	Response to ionizing radiation	0.005671
	Antigen processing and presentation of peptide antigen via MHC class I	0.007248
	Apoptosis	0.008696
	Antigen processing and presentation of peptide antigen	0.009478
	Programmed cell death	0.010298
	Cell death	0.010779
	Regulation of transcription	0.013117
	Death	0.013817
	Chromosome segregation	0.018543
	M phase of mitotic cell cycle	0.018583
	Response to radiation	0.021978
	Regulation of JNK cascade	0.022394
	Regulation of stress-activated protein kinase signaling pathway	0.024077
	Positive regulation of protein kinase activity	0.029813
	Mitotic cell cycle	0.033296
	Regulation of cellular response to stress	0.034011
	Positive regulation of kinase activity	0.037137
	Vesicle organization	0.037478
	Response to abiotic stimulus	0.039562
	M phase	0.040202
	DNA repair	0.040945
	Response to DNA damage stimulus	0.043932
	Positive regulation of transferase activity	0.044075

Table S4. *Cont.*

Category	GO_Term	*p*-Value
Biological Process	Transmission of nerve impulse	0.045742
	Proton-transporting ATP synthase complex assembly	0.04741
	Antigen processing and presentation of endogenous peptide antigen via MHC class I	0.04741
	Proton-transporting ATP synthase complex biogenesis	0.04741
Cellular Component	Non-membrane-bounded organelle	4.56×10^{-4}
	Intracellular non-membrane-bounded organelle	4.56×10^{-4}
	Condensed chromosome, centromeric region	5.54×10^{-4}
	Nucleolus	0.001334
	Chromosome, centromeric region	0.001389
	Intracellular organelle lumen	0.002934
	Organelle lumen	0.003094
	Kinetochore	0.003613
	Ribonucleoprotein complex	0.003792
	Membrane-enclosed lumen	0.005536
	Mitochondrial lumen	0.00595
	Mitochondrial matrix	0.00595
	Ribosome	0.006654
	Condensed chromosome kinetochore	0.008615
	Large ribosomal subunit	0.013807
	Condensed chromosome	0.014472
	Outer kinetochore of condensed chromosome	0.0146
	Mitochondrion	0.015452
	Mitochondrial ribosome	0.016379
	Organellar ribosome	0.016379
	Organellar large ribosomal subunit	0.016974
	Mitochondrial large ribosomal subunit	0.016974
	Ribosomal subunit	0.020892
	Anchored to membrane	0.027788

Table S4. *Cont.*

Category	GO_Term	*p*-Value
	Transition metal ion binding	2.29×10^{-6}
	Cation binding	1.26×10^{-5}
	Metal ion binding	1.35×10^{-5}
	Ion binding	1.42×10^{-5}
	Zinc ion binding	1.88×10^{-4}
	Structural constituent of ribosome	0.002423
Molecular Function	Cytokine receptor activity	0.008179
	Cytokine binding	0.01522
	Lanosterol *O*-acyltransferase activity	0.045087
	Ergosterol *O*-acyltransferase activity	0.045087
	Ribosylnicotinamide kinase activity	0.045087
	Sterol *O*-acyltransferase activity	0.045087
	Cholesterol *O*-acyltransferase activity	0.045087

MHC, major histocompatibility complex; J NK, Jun N-terminal kinase.

Table S5. Information of deregulated mRNAs by microarray analysis.

Probe Name	*p*-Value	Fold Change	Gene Symbol	Gene Name	Gene ID
A_51_P493987	0.001	52.94	*Gstm1*	glutathione S-transferase, mu 1	NM_010358
A_55_P2034531	0.000	48.48	*Cyp3a59*	cytochrome P450, subfamily 3A, polypeptide 59	NM_001105160
A_55_P2032081	0.001	28.51	*Cyp3a11*	cytochrome P450, family 3, subfamily a, polypeptide 11	NM_007818
A_55_P2032079	0.003	20.05	*Bglap2*	bone gamma-carboxyglutamate protein 2	NM_001032298
A_52_P63343	0.028	19.56	*Akr1c19*	aldo-keto reductase family 1, member C19	NM_001013785
A_51_P389539	0.001	13.82	*Rdh11*	retinol dehydrogenase 11	NM_021557
A_51_P484526	0.000	10.12	*Cyp51*	cytochrome P450, family 51	NM_020010
A_52_P235347	0.007	9.58	*Cxcl13*	chemokine (C-X-C motif) ligand 13	NM_018866
A_51_P249286	0.028	7.88	*Gstm3*	glutathione S-transferase, mu 3	NM_010359
A_51_P397934	0.000	7.83	*Pvt1*	plasmacytoma variant translocation 1	NR_003368
A_55_P1985544	0.000	7.10	*Acot6*	acyl-CoA thioesterase 6	ENSMUST00000056822
A_55_P2033600	0.000	6.75	*Mtnr1a*	melatonin receptor 1A	NM_008639
A_52_P229052	0.000	6.45	*Dio1*	deiodinase, iodothyronine, type I	NM_007860
A_51_P320452	0.005	6.22	*Cidea*	cell death-inducing DNA fragmentation factor, alpha subunit-like effector A	NM_007702
A_51_P156955	0.037	4.94	*Mgll*	monoglyceride lipase	NM_001166250

Table S5. *Cont.*

Probe Name	*p*-Value	Fold Change	Gene Symbol	Gene Name	Gene ID
A_66_P106611	0.000	4.51	*Slc2a4*	solute carrier family 2 (facilitated glucose transporter), member 4	NM_009204
A_51_P327491	0.013	4.36	*Gpr98*	G protein-coupled receptor 98	NM_054053
A_51_P296608	0.009	4.20	*Nat8*	*N*-acetyltransferase 8 (GCN5-related, putative)	NM_023455
A_55_P2293668	0.004	3.86	*Sprr1a*	small proline-rich protein 1A	NM_009264
A_55_P2136561	0.013	3.70	*Hmgcs1*	3-hydroxy-3-methylglutaryl-Coenzyme A synthase 1	NM_145942
A_55_P2135203	0.000	3.63	*Mirg*	miRNA containing gene	AK077315
A_55_P2185178	0.003	3.60	*Rnf125*	ring finger protein 125	NM_026301
A_55_P2121956	0.042	3.60	*Dct*	dopachrome tautomerase	NM_010024
A_51_P217498	0.049	3.57	*Rgs16*	regulator of G-protein signaling 16	NM_011267
A_52_P204311	0.002	3.53	*Ppp1r3g*	protein phosphatase 1, regulatory (inhibitor) subunit 3G	NM_029628
A_51_P282760	0.030	3.50	*Cyp3a41b*	cytochrome P450, family 3, subfamily a, polypeptide 41B	NM_001105159
A_55_P2170881	0.001	3.28	*Figf*	c-fos induced growth factor	NM_010216
A_51_P139678	0.003	3.22	*Ccbp2*	chemokine binding protein 2	NM_021609
A_55_P2082215	0.000	3.22	*Acmsd*	amino carboxymuconate semialdehyde decarboxylase	NM_001033041
A_52_P164136	0.029	3.18	*Slc13a5*	solute carrier family 13 (sodium-dependent citrate transporter), member 5	NM_001004148
A_55_P2135153	0.012	3.18	*Gngt1*	guanine nucleotide binding protein (G protein), gamma transducing activity polypeptide 1	NM_010314
A_55_P2157093	0.002	3.15	*0610010O12Rik*	RIKEN cDNA 0610010O12 gene	ENSMUST00000050584
A_55_P2387665	0.002	3.07	*Slc22a7*	solute carrier family 22 (organic anion transporter), member 7	NM_144856
A_51_P456208	0.011	3.00	*Idi1*	isopentenyl-diphosphate delta isomerase	NM_145360
A_55_P2022074	0.004	2.96	*Neu3*	neuraminidase 3	NM_016720
A_51_P357735	0.008	2.94	*Trem2*	triggering receptor expressed on myeloid cells 2	NM_031254
A_51_P313581	0.000	2.90	*Slc22a29*	solute carrier family 22. member 29	NM_172776
A_51_P387400	0.003	2.84	*Aatk*	apoptosis-associated tyrosine kinase	NM_007377
A_55_P2042076	0.000	2.84	*Bglap-rs1*	bone gamma-carboxyglutamate protein, related sequence 1	NM_031368
A_66_P136186	0.048	2.81	*Fam89a*	family with sequence similarity 89, member A	NM_001081120
A_55_P2120919	0.036	2.80	*Tef*	thyrotroph embryonic factor	NM_017376
A_51_P144531	0.033	2.78			ENSMUST00000094754
A_51_P276943	0.000	2.77	*Dkk4*	dickkopf homolog 4 (*Xenopus laevis*)	NM_145592
A_51_P287198	0.005	2.75			ENSMUST00000095071
A_55_P2037787	0.046	2.74	*Cabyr*	calcium-binding tyrosine-(Y)-phosphorylation regulated (fibrousheathin 2)	NM_027687
A_51_P455338	0.001	2.74	*Pmvk*	phosphomevalonate kinase	NM_026784
A_55_P1979893	0.012	2.71	*Fabp5*	fatty acid binding protein 5, epidermal	NM_010634
A_55_P2042156	0.005	2.67	*Cd207*	CD207 antigen	NM_144943
A_51_P403477	0.005	2.63	*2810408I11Rik*	RIKEN cDNA 2810408I11 gene	NR_038009

Table S5. *Cont.*

Probe Name	*p*-Value	Fold Change	Gene Symbol	Gene Name	Gene ID
A_51_P243755	0.002	2.63	*Hunk*	hormonally upregulated Neu-associated kinase	NM_015755
A_66_P122086	0.004	2.62	*Wee1*	Wee 1 homolog 1 (*Schizosaccharomyces pombe*)	NM_009516
A_55_P2039429	0.001	2.62	*Serpina12*	serine (or cysteine) peptidase inhibitor, clade A (alpha-1 antiproteinase, antitrypsin), member 12	NM_026535
A_55_P2159934	0.015	2.60	*Cyp3a11*	cytochrome P450, family 3, subfamily a, polypeptide 11	NM_007818
A_52_P289091	0.004	2.59	*Rnf186*	ring finger protein 186	NM_025786
A_51_P199168	0.010	2.54	*Acot1*	acyl-CoA thioesterase 1	NM_012006
A_55_P2119257	0.041	2.53	*Chrna4*	cholinergic receptor, nicotinic, alpha polypeptide 4	NM_015730
A_52_P163021	0.001	2.52	*Adamdec1*	Adamlike, decysin 1	NM_021475
A_55_P2078830	0.036	2.51	*Cyp7a1*	cytochrome P450, family 7, subfamily a, polypeptide 1	NM_007824
A_55_P1994112	0.003	2.49	*Slc13a5*	solute carrier family 13 (sodium-dependent citrate transporter), member 5	NM_001004148
A_55_P2096043	0.001	2.47	*Arrdc3*	arrestin domain containing 3	NM_001042591
A_55_P2083894	0.034	2.46	*Dhcr7*	7-dehydrocholesterol reductase	NM_007856
A_55_P2027102	0.048	2.44	*Lonrf3*	Lonpeptidase N-terminal domain and ring finger 3	NM_028894
A_55_P1953341	0.043	2.40	*Apol7b*	apolipoprotein L 7b	NM_001024848
A_51_P258493	0.023	2.39			NAP002856-002
A_55_P2034067	0.034	2.39	*Arhgap26*	Rho GTPase activating protein 26	NM_175164
A_55_P2002557	0.000	2.38	*Gstm6*	glutathione *S*-transferase, mu 6	NM_008184
A_51_P395856	0.000	2.38			
A_55_P2075263	0.046	2.37	*Tbc1d30*	TBC1 domain family, member 30	NM_029057
A_55_P2114863	0.002	2.35	*Slc17a8*	solute carrier family 17 (sodium-dependent inorganic phosphate cotransporter), member 8	NM_182959
A_55_P2063316	0.001	2.31	*Atg16l2*	autophagy related 16 like 2 (*Saccharomycecerevisiae*)	NM_001111111
A_55_P1963508	0.010	2.30	*Cyp4a12a*	cytochrome P450, family 4, subfamily a, polypeptide 12a	NM_177406
A_51_P346893	0.001	2.29	*Prss8*	protease, serine, 8 (prostasin)	NM_133351
A_55_P2165091	0.039	2.29	*Rnf144a*	ring finger protein 144A	NM_080563
A_55_P1986247	0.010	2.27	*Atg16l2*	autophagy related 16 like 2 (*S. cerevisiae*)	NM_001111111
A_55_P1962937	0.006	2.25			NAP111439-1
A_51_P223776	0.000	2.22	*Cyp2c67*	cytochrome P450, family 2, subfamily c, polypeptide 67	NM_001024719
A_55_P2046657	0.006	2.20			
A_55_P2130885	0.017	2.17	*Gpc1*	glypican 1	NM_016696
A_55_P1973844	0.015	2.12	*Gm3776*	predicted gene 3776	NM_001243092
A_66_P130115	0.030	2.12	*Abtb2*	ankyrin repeat and BTB domain containing 2	NM_178890
A_66_P127161	0.001	2.11	*Arntl*	aryl hydrocarbon receptor nuclear translocator-like	NM_007489
A_55_P2054342	0.002	2.09	*Grk5*	G protein-coupled receptor kinase 5	NM_018869
A_51_P269792	0.000	2.08	*Lpl*	lipoprotein lipase	NM_008509

Table S5. *Cont.*

Probe Name	*p*-Value	Fold Change	Gene Symbol	Gene Name	Gene ID
A_52_P315976	0.007	2.08	*Fut1*	fucosyltransferase 1	NM_008051
A_55_P2142072	0.004	2.07	*Raet1c*	retinoic acid early transcript gamma	NM_009018
A_52_P656845	0.009	2.06	*Cyp4a31*	cytochrome P450, family 4, subfamily a, polypeptide 31	NM_001252539
A_55_P2044242	0.002	2.05	*Lss*	lanosterol synthase	NM_146006
A_55_P2063312	0.001	2.05	*Cyfip2*	cytoplasmic FMR1 interacting protein 2	NM_001252460
A_51_P272553	0.025	2.04	*Ttyh1*	tweety homolog 1 (*Drosophila melanogaster*)	NM_021324
A_66_P135391	0.022	0.50	*Pdzk1ip1*	PDZK1 interacting protein 1	NM_001164557
A_51_P386539	0.037	0.49	*Cables1*	CDK5 and Abl enzyme substrate 1	NM_022021
A_55_P2056729	0.029	0.49	*4933437F05Rik*	RIKEN cDNA 4933437F05 gene	NM_027744
A_55_P2031671	0.015	0.49	*Per3*	period homolog 3 (*D. melanogaster*)	NM_011067
A_51_P515965	0.046	0.49	*Nsdhl*	NAD(P) dependent steroid dehydrogenase-like	NM_010941
A_66_P105422	0.000	0.49	*Extl1*	exostoses (multiple)-like 1	NM_019578
A_55_P2072091	0.005	0.49	*Raet1c*	retinoic acid early transcript gamma	NM_009018
A_55_P2014882	0.007	0.49	*Klf10*	Kruppel-like factor 10	NM_013692
A_52_P227937	0.006	0.49	*Cyp2c68*	cytochrome P450, family 2, subfamily c, polypeptide 68	NM_001039555
A_51_P117739	0.000	0.49	*Gm2814*	predicted gene 2814	AK144055
A_66_P100419	0.000	0.49	*Ddc*	dopa decarboxylase	NM_016672
A_55_P2150876	0.004	0.48	*Meg3*	maternally expressed 3	NR_027652
A_55_P2198983	0.004	0.48	*Sqle*	squalene epoxidase	NM_009270
A_55_P2032966	0.005	0.48	*Cfd*	complement factor D (adipsin)	NM_013459
A_51_P327585	0.014	0.48	*9130409I23Rik*	RIKEN cDNA 9130409I23 gene	NM_001033819
A_52_P222073	0.000	0.48	*Tmem218*	transmembrane protein 218	NM_025464
A_55_P2130535	0.022	0.48	*Cyp4a14*	cytochrome P450, family 4, subfamily a, polypeptide 14	NM_007822
A_55_P1962661	0.000	0.48	*Rgs3*	regulator of G-protein signaling 3	NM_134257
A_55_P1966432	0.004	0.48	*Als2cr12*	amyotrophic lateral sclerosis 2 (juvenile) chromosome region, candidate 12 (human)	NM_175370
A_55_P1963692	0.008	0.48	*Lpl*	lipoprotein lipase	NM_008509
A_51_P155196	0.041	0.47	*Mgll*	monoglyceride lipase	NM_001166251
A_52_P63905	0.000	0.47	*Trhde*	TRH-degrading enzyme	NM_146241
A_55_P1952399	0.002	0.47	*Gm10639*	predicted gene 10639	NM_001122660
A_51_P479818	0.003	0.47	*Cyp2b13*	cytochrome P450, family 2, subfamily b, polypeptide 13	NM_007813
A_55_P1969575	0.020	0.47	*Dio1*	deiodinase, iodothyronine, type I	NM_007860
A_51_P350048	0.007	0.47	*Sema5b*	sema domain, seven thrombospondin repeats (type 1 and type 1-like), transmembrane domain (TM) and short cytoplasmic domain, (semaphorin) 5B	NM_013661
A_55_P2110497	0.005	0.46	*Serpina5*	serine (or cysteine) peptidase inhibitor, clade A, member 5	NM_172953
A_55_P1977144	0.000	0.46	*Igfbp2*	insulin-like growth factor binding protein 2	NM_008342

Table S5. *Cont.*

Probe Name	*p*-Value	Fold Change	Gene Symbol	Gene Name	Gene ID
A_51_P191354	0.000	0.46	*Srebf1*	sterol regulatory element binding transcription factor 1	NM_011480
A_55_P2065231	0.015	0.46	*Nsdhl*	NAD(P) dependent steroid dehydrogenase-like	NM_010941
A_55_P1997837	0.009	0.46	*Tmprss4*	transmembrane protease, serine 4	NM_145403
A_55_P2022337	0.016	0.46	*Cyp2u1*	cytochrome P450, family 2, subfamily u, polypeptide 1	NM_027816
A_55_P2028961	0.021	0.46	*Ear11*	eosinophil-associated, ribonuclease A family, member 11	NM_053113
A_55_P2332194	0.028	0.46	*Dct*	dopachrome tautomerase	NM_010024
A_55_P1989673	0.000	0.46	*Ddc*	dopa decarboxylase	NM_001190448
A_51_P350453	0.030	0.46			AI451831
A_51_P444954	0.021	0.45	*Dnmt3b*	DNA methyltransferase 3B	NM_001003961
A_55_P2086507	0.029	0.45	*Acot11*	acyl-CoA thioesterase 11	NM_025590
A_66_P110769	0.010	0.45	*Fdft1*	farnesyl diphosphate farnesyl transferase 1	NM_010191
A_51_P454196	0.002	0.45	*Cyp2g1*	cytochrome P450, family 2, subfamily g, polypeptide 1	NM_013809
A_55_P1985428	0.000	0.45	*Mgll*	monoglyceride lipase	NM_001166251
A_55_P2058433	0.000	0.45	*Pdk4*	pyruvate dehydrogenase kinase, isoenzyme 4	NM_013743
A_55_P2012478	0.021	0.45	*Dio1*	deiodinase, iodothyronine, type I	NM_007860
A_51_P142923	0.027	0.45	*Bhlhe40*	basic helix-loop-helix family, member e40	NM_011498
A_55_P2161347	0.001	0.44	*Slc10a2*	solute carrier family 10, member 2	NM_011388
A_55_P2033250	0.005	0.44	*Bcl2l14*	Bcl-like 14 (apoptosis facilitator)	NM_025778
A_52_P84027	0.034	0.44	*Ndrg1*	N-myc downstream regulated gene 1	NM_008681
A_52_P562267	0.001	0.44	*Gstm2*	glutathione *S*-transferase, mu 2	NM_008183
A_55_P2100928	0.011	0.44	*Dhrs9*	dehydrogenase/reductase (SDR family) member 9	NM_175512
A_55_P2039044	0.014	0.44	*Fabp2*	fatty acid binding protein 2, intestinal	NM_007980
A_55_P1956448	0.001	0.43	*Cyp3a16*	cytochrome P450, family 3, subfamily a, polypeptide 16	NM_007820
A_55_P2009952	0.022	0.43	*Agxt2l1*	alanine-glyoxylate aminotransferase 2-like 1	NM_027907
A_51_P245368	0.006	0.43	*Cyp4a12b*	cytochrome P450, family 4, subfamily a, polypeptide 12B	NM_172306
A_51_P247637	0.020	0.43	*9130221J18Rik*	RIKEN cDNA 9130221J18 gene	AK033690
A_55_P2051254	0.000	0.43	*Gm10701*	predicted gene 10701	AK144276
A_55_P2013586	0.002	0.43	*Cyp2c55*	cytochrome P450, family 2, subfamily c, polypeptide 55	NM_028089
A_52_P515036	0.000	0.43	*Serpine1*	serine (or cysteine) peptidase inhibitor, clade E, member 1	NM_008871
A_55_P2011678	0.005	0.43			
A_55_P2038358	0.001	0.43	*Acss2*	acyl-CoA synthetase short-chain family member 2	NM_019811
A_55_P1961466	0.004	0.43	*Gal3st1*	galactose-3-*O*-sulfotransferase 1	NM_016922
A_55_P2038987	0.001	0.42	*Hmgcs1*	3-hydroxy-3-methylglutaryl-Coenzyme A synthase 1	AK031297
A_51_P209372	0.029	0.42	*BC057022*	cDNA sequence BC057022	NM_001004180
A_51_P331207	0.002	0.42	*Wfdc2*	WAP four-disulfide core domain 2	NM_026323

Table S5. *Cont.*

Probe Name	*p*-Value	Fold Change	Gene Symbol	Gene Name	Gene ID
A_55_P2017362	0.005	0.42	*Rad51l1*	RAD51-like 1 (S. cerevisiae)	NM_009014
A_66_P111660	0.049	0.42	*Fam89a*	family with sequence similarity 89, member A	NM_001081120
A_55_P2360266	0.036	0.42	*Srgap3*	SLIT-ROBO Rho GTPase activating protein 3	NM_080448
A_52_P164161	0.006	0.42	*AI132709*	expressed sequence AI132709	AI266897
A_52_P197402	0.018	0.42			NAP111402-1
A_55_P2019113	0.000	0.41	*Slc39a4*	solute carrier family 39 (zinc transporter), member 4	NM_028064
A_55_P2107070	0.023	0.41	*Igfbp2*	insulin-like growth factor binding protein 2	NM_008342
A_52_P512553	0.001	0.41	*Zfp618*	zinc fingerprotein 618	NM_028326
A_51_P164296	0.003	0.41	*Chka*	choline kinase alpha	NM_013490
A_55_P2046411	0.017	0.41	*Tpm2*	tropomyosin 2, beta	NM_009416
A_55_P1976395	0.013	0.41	*Mt1*	metallothionein 1	NM_013602
A_51_P112223	0.000	0.41	*Gas7*	growth arrest specific 7	NM_001109657
A_52_P141488	0.045	0.41	*Lcn13*	lipocalin 13	NM_153558
A_51_P266618	0.000	0.41	*Gck*	glucokinase	NM_010292
A_55_P2003053	0.004	0.40	*Mas1*	MAS1 oncogene	NM_008552
A_51_P296487	0.004	0.40	*Sh2d4a*	SH2 domain containing 4A	NM_028182
A_52_P140005	0.021	0.40	*Pmvk*	phosphomevalonate kinase	NM_026784
A_55_P1954569	0.016	0.40	*Aldoc*	aldolase C, fructose-bisphosphate	NM_009657
A_51_P320852	0.002	0.40	*9030619P08Rik*	RIKEN cDNA 9030619P08 gene	NM_001039720
A_55_P2007919	0.004	0.40	*Inhbe*	inhibin beta E	NM_008382
A_55_P2177410	0.019	0.40	*Nfe2*	nuclear factor, erythroid derived 2	NM_008685
A_51_P383774	0.012	0.40	*Gm10639*	predicted gene 10639	NM_001122660
A_55_P2214124	0.022	0.39	*Acnat2*	acyl-coenzyme A amino acid N-acyltransferase 2	NM_145368
A_52_P357611	0.005	0.39	*Fmn2*	formin 2	NM_019445
A_55_P1967538	0.004	0.39	*Cyp3a59*	cytochrome P450, subfamily 3A, polypeptide 59	NM_001105160
A_51_P137452	0.000	0.39	*Spry4*	sprouty homolog 4 (*D. melanogaster*)	NM_011898
A_51_P117581	0.003	0.39	*Tuba8*	tubulin, alpha 8	NM_017379
A_55_P2171413	0.019	0.39			
A_55_P2143923	0.006	0.39			ENSMUST00000098589
A_55_P2028046	0.000	0.39	*Cyp8b1*	cytochrome P450, family 8, subfamily b, polypeptide 1	NM_010012
A_51_P505662	0.001	0.38	*Cyp4a30b-ps*	cytochrome P450, family 4, subfamily a, polypeptide 30b, pseudogene	NM_001100185
A_55_P1966438	0.039	0.38	*1110006E14Rik*	RIKEN cDNA 1110006E14 gene	AK141773
A_51_P391616	0.000	0.38	*4833411C07Rik*	RIKEN cDNA 4833411C07 gene	BC052524
A_52_P257812	0.002	0.38	*Tmem28*	transmembrane protein 28	ENSMUST00000096363
A_52_P24690	0.018	0.37	*LOC100502818*	uncharacterized LOC100502818	XM_003084779

Table S5. *Cont.*

Probe Name	*p*-Value	Fold Change	Gene Symbol	Gene Name	Gene ID
A_55_P2140031	0.002	0.37	*Acss2*	acyl-CoA synthetase short-chain family member 2	NM_019811
A_55_P1989981	0.001	0.37	*Fam171b*	family with sequence similarity 171, member B	NM_175514
A_55_P2150976	0.014	0.37	*Igh-VJ558*	immunoglobulin heavy chain (J558 family)	BC019425
A_55_P2070992	0.004	0.37	*Gsta4*	glutathione S-transferase, alpha 4	NM_010357
A_51_P393654	0.030	0.37	*Htra4*	HtrA serine peptidase 4	NM_001081187
A_52_P251623	0.026	0.37	*Grin3b*	glutamate receptor, ionotropic, NMDA3B	NM_130455
A_55_P2047809	0.000	0.36	*Osbp2*	oxysterol binding protein 2	NM_152818
A_51_P405606	0.008	0.36	*Snhg11*	small nucleolar RNA host gene 11	NM_175692
A_51_P259296	0.001	0.36	*Gm129*	predicted gene 129	NM_001033302
A_55_P2026761	0.014	0.36	*Gm10804*	predicted gene 10804	NR_040533
A_55_P1953387	0.013	0.36			
A_55_P2043627	0.003	0.36	*Dbp*	D site albumin promoter binding protein	NM_016974
A_55_P2122841	0.012	0.35	*Gna14*	guanine nucleotide binding protein, alpha 14	NM_008137
A_55_P1966804	0.016	0.35			
A_55_P1996702	0.001	0.35	*Tmeff2*	transmembrane protein with EGF-like and two follistatin-like domains 2	NM_019790
A_55_P2152607	0.000	0.35	*Slco2a1*	solute carrier organic anion transporter family, member 2a1	NM_033314
A_55_P2168267	0.018	0.35	*Snhg11*	small nucleolar RNA host gene 11	NM_175692
A_55_P2076533	0.006	0.34	*Dbp*	D site albumin promoter binding protein	NM_016974
A_55_P1962771	0.000	0.34	*Krt23*	keratin 23	NM_033373
A_55_P1972322	0.002	0.34	*Nipal1*	NIPA-like domain containing 1	NM_001081205
A_55_P2043083	0.000	0.34	*Asns*	asparagine synthetase	NM_012055
A_52_P539161	0.009	0.34	*Gpr110*	G protein-coupled receptor 110	NM_133776
A_66_P137462	0.018	0.34	*Cabyr*	calcium-binding tyrosine-(Y)-phosphorylation regulated (fibrousheathin 2)	NM_027687
A_51_P479230	0.000	0.34	*Cyp2c67*	cytochrome P450, family 2, subfamily c, polypeptide 67	NM_001024719
A_52_P207314	0.008	0.34	*Gm5589*	predicted gene 5589	BC080727
A_55_P1988789	0.016	0.34	*Lonrf3*	Lonpeptidase N-terminal domain and ring finger 3	NM_028894
A_55_P2005426	0.016	0.34	*Gstm3*	glutathione S-transferase, mu 3	NM_010359
A_51_P110381	0.003	0.33	*Gadd45a*	growth arrest and DNA-damage-inducible 45 alpha	NM_007836
A_55_P1988795	0.007	0.33			CA492558
A_55_P2099840	0.010	0.33	*Sc4mol*	sterol-C4-methyl oxidase-like	NM_025436
A_55_P2081116	0.003	0.33	*Moxd1*	monooxygenase, DBH-like 1	NM_021509
A_51_P103222	0.000	0.32	*Fdps*	farnesyl diphosphate synthetase	NM_001253751
A_66_P121059	0.001	0.32			NAP114472-1
A_55_P2178578	0.014	0.32	*Meg3*	maternally expressed 3	NR_027652
A_55_P2013823	0.001	0.32	*Gm15998*	predicted gene 15998	XR_104847

Table S5. *Cont.*

Probe Name	*p*-Value	Fold Change	Gene Symbol	Gene Name	Gene ID
A_52_P566840	0.008	0.32	*Acnat2*	acyl-coenzyme A amino acid *N*-acyltransferase 2	NM_145368
A_51_P492410	0.007	0.32	*Loxl4*	lysyl oxidase-like 4	NM_001164311
A_52_P2710	0.000	0.32	*Fgf21*	fibroblast growth factor 21	NM_020013
A_51_P411345	0.002	0.31	*Meig1*	meiosis expressed gene 1	NM_008579
A_55_P2106803	0.044	0.31	*Fam129b*	family with sequence similarity 129, member B	NM_146119
A_66_P107231	0.023	0.29	*Mirg*	miRNA containing gene	NR_028265
A_55_P2408588	0.033	0.29			
A_55_P2178800	0.000	0.29	*Gsta2*	glutathione S-transferase, alpha 2 (Yc2)	NM_008182
A_66_P124179	0.024	0.28	*Myom3*	myomesin family, member 3	NM_001085509
A_51_P238576	0.021	0.28	*Ptgds*	prostaglandin D2 synthase (brain)	NM_008963
A_55_P2121225	0.000	0.26	*Wif1*	Wnt inhibitory factor 1	NM_011915
A_55_P1975185	0.013	0.26	*Raet1e*	retinoic acid early transcript 1E	NM_198193
A_55_P1957918	0.007	0.25	*Stap1*	signal transducing adaptor family member 1	NM_019992
A_51_P267933	0.000	0.25	*Btg3*	B cell translocation gene 3	NM_009770
A_55_P2046408	0.003	0.24	*Ttbk1*	tau tubulin kinase 1	NM_001162864
A_55_P2115955	0.019	0.23	*Fam19a2*	family with sequence similarity 19, member A2	NM_182807
A_51_P355301	0.000	0.22	*Tff3*	trefoil factor 3, intestinal	NM_011575
A_55_P2148071	0.000	0.22	*Gstm4*	glutathione *S*-transferase, mu 4	NM_026764
A_51_P482051	0.000	0.22	*Gstt2*	glutathione *S*-transferase, theta 2	NM_010361
A_51_P268529	0.003	0.21	*Atp6v0d2*	ATPase, H+ transporting, lysosomal V0 subunit D2	NM_175406
A_55_P1959748	0.013	0.20			AK038006
A_66_P106148	0.010	0.18	*Cd9*	CD9 antigen	NM_007657
A_55_P2001780	0.000	0.18	*Grm8*	glutamate receptor, metabotropic 8	NM_008174
A_51_P447785	0.000	0.17	*Per2*	period homolog 2 (*D. melanogaster*)	NM_011066
A_51_P378789	0.000	0.17	*Fam129b*	family with sequence similarity 129, member B	NM_146119
A_51_P469951	0.001	0.16	*Nr1d1*	nuclear receptor subfamily 1, group D, member 1	NM_145434
A_55_P2164075	0.003	0.16	*Slc13a2*	solute carrier family 13 (sodium-dependent dicarboxylate transporter), member 2	NM_022411
A_55_P2102060	0.006	0.16	*Htatip2*	HIV-1 tat interactive protein 2, homolog (human)	NM_016865
A_55_P2033407	0.008	0.16	*Apoa4*	apolipoprotein A-IV	NM_007468
A_52_P350750	0.046	0.15	*Mogat2*	monoacylglycerol *O*-acyltransferase 2	NM_177448
A_55_P2170454	0.001	0.15	*BC068157*	cDNA sequence BC068157	NM_207203
A_51_P155873	0.035	0.14	*Cml5*	camello-like 5	NM_023493
A_51_P455997	0.007	0.14	*Fads2*	fatty acid desaturase 2	NM_019699
A_55_P2102065	0.001	0.14	*Synj2*	synaptojanin 2	NM_011523
A_55_P2069969	0.000	0.13	*Meig1*	meiosis expressed gene 1	NM_008579

Table S5. *Cont.*

Probe Name	*p*-Value	Fold Change	Gene Symbol	Gene Name	Gene ID
A_55_P2336173	0.003	0.13	*Me1*	malic enzyme 1, NADP(+)-dependent, cytosolic	NM_001198933
A_55_P1962516	0.005	0.13	*4930415C11Rik*	RIKEN cDNA 4930415C11 gene	AI606402
A_55_P2093705	0.000	0.13	*Csad*	cysteine sulfinic acid decarboxylase	NM_144942
A_55_P2093704	0.001	0.13	*Asap2*	ArfGAP with SH3 domain, ankyrin repeat and PH domain 2	NM_001135192
A_66_P119518	0.000	0.12	*Me1*	malic enzyme 1, NADP(+)-dependent, cytosolic	NM_001198933
A_55_P2032946	0.001	0.12	*Abcb1b*	ATP-binding cassette, sub-family B (MDR/TAP), member 1B	NM_011075
A_55_P2058550	0.007	0.11	*Sema5b*	sema domain, seven thrombospondin repeats (type 1 and type 1-like), transmembrane domain (TM) and short cytoplasmic domain, (semaphorin) 5B	NM_013661
A_55_P2017116	0.009	0.11	*Ugt1a10*	UDP glycosyltransferase 1 family, polypeptide A10	NM_201641
A_55_P2419299	0.002	0.08	*Kcnk10*	potassium channel, subfamily K, member 10	NM_029911

BTB, broad-complex, tramtrack, and bric-à-brac; FMR, fragile X mental retardation; PDZK, PDZ-containing kidney protein; TRH, thyrotropin releasing hormone; WAP, whey acidic protein; EGF, epidermal growth factor; NIPA, nuclear interaction partner of anaplastic lymphoma receptor tyrosine kinase; DBH, dopamine β-hydroxylase

Table S6. GO pathway categories of potential *cis*/*trans*-targets from significantly upregulated and downregulated lncRNAs in an NAFLD animal model.

Category	GO_Term	*p*-Value
**Upregulated mRNAs:**		
Biological Process	Oxidation reduction	1.40×10^{-11}
	Sterol biosynthetic process	$2.15E \times 10^{-8}$
	Lipid biosynthetic process	5.70×10^{-8}
	Cholesterol biosynthetic process	1.11×10^{-8}
	Sterol metabolic process	1.15×10^{-7}
	Cholesterol metabolic process	7.10×10^{-7}
	Steroid biosynthetic process	7.93×10^{-11}
	Steroid metabolic process	1.11×10^{-6}
	Circadian rhythm	2.26×10^{-6}
	Rhythmic process	8.03×10^{-5}
	Isoprenoid biosynthetic process	0.001549
	Isoprenoid metabolic process	0.001771
	Fatty acid metabolic process	0.003451
	Secondary metabolic process	0.006139
	Organic anion transport	0.00806
	Cellular amino acid derivative metabolic process	0.017095
	Carboxylic acid biosynthetic process	0.017095
	Organic acid biosynthetic process	0.017095
	Coenzyme metabolic process	0.018058
Cellular Component	Microsome	1.09×10^{-12}
	Vesicular fraction	1.89×10^{-12}
	Endoplasmic reticulum	2.58×10^{-8}
	Cell fraction	6.03×10^{-8}
	Membrane fraction	9.84×10^{-8}
	Insoluble fraction	1.73×10^{-7}
	Extrinsic to membrane	5.42×10^{-5}
	Extracellular region	0.005526

Table S6. *Cont.*

Category	GO_Term	*p*-Value
Molecular Function	Heme binding	7.60×10^{-12}
	Tetrapyrrole binding	1.58×10^{-11}
	Glutathione transferase activity	3.20×10^{-10}
	Oxidoreductase activity	1.33×10^{-9}
	Transferase activity, transferring alkyl or aryl (other than methyl) groups	7.63×10^{-9}
	Electron carrier activity	1.02×10^{-8}
	Aromatase activity	9.26×10^{-8}
	Iron ion binding	3.91×10^{-7}
	Acyl-CoA thioesterase activity	0.021581
	CoA hydrolase activity	0.032835
	Carboxylesterase activity	0.035344
	Carboxy-lyase activity	0.037853

Table S6. *Cont.*

Category	GO_Term	*p*-Value
**Downregulated mRNAs:**		
Biological Process	Oxidation reduction	3.96×10^{-12}
	Sterol biosynthetic process	1.25×10^{-9}
	Lipid biosynthetic process	1.32×10^{-9}
	Cholesterol biosynthetic process	9.73×10^{-9}
	Steroid biosynthetic process	3.41×10^{-8}
	Sterol metabolic process	6.51×10^{-8}
	Steroid metabolic process	1.94×10^{-7}
	Cholesterol metabolic process	5.87×10^{-7}
	Isoprenoid metabolic process	3.89×10^{-4}
	Isoprenoid biosynthetic process	4.77×10^{-4}
	Secondary metabolic process	0.012791
	Carboxylic acid biosynthetic process	0.017699
	Organic acid biosynthetic process	0.017699
	Cellular amino acid derivative metabolic process	0.017699
	Terpenoid metabolic process	0.017718
	Fatty acid metabolic process	0.04134
	Benzene and derivative metabolic process	0.041656
	Acetyl-CoA biosynthetic process	0.041656
	Phosphatidylcholine biosynthetic process	0.04843
	Cellular zinc ion homeostasis	0.04843
Cellular Component	Microsome	2.77×10^{-12}
	Vesicular fraction	4.38×10^{-12}
	Endoplasmic reticulum	1.92×10^{-8}
	Membrane fraction	4.00×10^{-7}
	Cell fraction	5.28×10^{-7}
	Insoluble fraction	6.22×10^{-7}
	Extrinsic to membrane	2.87×10^{-5}

Table S6. *Cont.*

Category	GO_Term	*p*-Value
Molecular Function	Glutathione transferase activity	9.96×10^{-12}
	Heme binding	4.58×10^{-11}
	Tetrapyrrole binding	8.36×10^{-11}
	Transferase activity, transferring alkyl or aryl (other than methyl) groups	1.65×10^{-10}
	Electron carrier activity	3.09×10^{-12}
	Oxidoreductase activity	3.14×10^{-8}
	Aromatase activity	1.67×10^{-7}
	Iron ion binding	2.34×10^{-7}
	Carboxy-lyase activity	0.017087
	Intramolecular oxidoreductase activity	0.032464
	Alcohol dehydrogenase (NAD) activity	0.043886

Table S7. Primers of the genes selected for qPCR analysis.

Name	Description/Probe Name	GenBank/Location	Primer Sequence (5′–3′)
**mRNA:**			
Arntl	Aryl hydrocarbon receptor nuclear translocator-like	NM_007489	F: TCATGAGCCTCTTGGAAGCA<br>R: GGCCATGGCAAGTCACTAAAG
Per2	Pperiod homolog 2 (*D. melanogaster*)	NM_011066	F: TGCTGGTCCTCAGATCACTG<br>R: TAGAGCTGGGCAGAGAGGTC
Per3	Period homolog 3 (*D. melanogaster*)	NM_011067	F: CTTTTCACCAAACGACACGA<br>R: CTCATGGGACTCTGCTGTCA
Nr1d1	Nuclear receptor subfamily 1, group D, member 1	NM_145434	F: CCCAACGACAACAACCTTTT<br>R: GTGCCCATTGCTGTTAGGTT
Fads2	Fatty acid desaturase 2	NM_019699	F: CCTTTGTCCTCGCTACCTCTCA<br>R: ATAGACAGAAAGGTGGCCATAGTCA
Lpl	Lipoprotein lipase	NM_008509	F: GGTATCTGAACACCAACCCACAT<br>R: TATCCCTTGACTCTTTCCTTTCTGA
Fabp5	Fatty acid binding protein 5, epidermal	NM_010634	F: TGTCATGAACAATGCCACCT<br>R: CTGGCAGCTAACTCCTGTCC

Table S7. *Cont.*

Name	Description/Probe Name	GenBank/Location	Primer Sequence (5′–3′)
Asap2	Arf GAP with SH3 domain, ankyrin repeat and PH domain 2	NM_001135192	F: ACGCATCTGTATCATCGGTCTTAG<br>R: GGGACTGGAGTTAAAATGTTGACA
Acmsd	Amino carboxymuconate semialdehyde decarboxylase	NM_001033041	F: CCTGAGTCTGGAAGGATGGA<br>R: GCCTCAAACACAGACCCATT
Actin	Beta-actin	NM_007392	F: TGGCTCCTAGCACCATGAAGA<br>R: GCCACCGATCCACACAGAGT
**lncRNA:**			
FLRL1	CUST_16222_PI428753551	chr4(151033075, 151036561, −)	F: CCCATCTCTGTTGGTGACCT<br>R: CGGCAAACTCAACCATTCTT
FLRL2	CUST_39241_PI42875355	chr7(113240249, 113243748, +)	F: TGGTGTGTGTGAGCCTGAGAA<br>R: AGCAGCAAGTAATAGGATGAGCAA
FLRL3	CUST_56439_PI428753551	chr4(11611139, 11615007, −)	F: CTGGAGGGAAGACGATCGAA<br>R: TGCGAGCTGGTTCTGTAACATT
FLRL4	CUST_6576_PI428753551	chr12(21269099, 21270170, +)	F: CACGCATCTGTATCATCGGTCTT<br>R: GGGACTGGAGTTAAAATGTTGACA
FLRL5	CUST_69671_PI428753551	chr9(46239840, 46242032, −)	F: GGCCACCTGGTCCGAAGT<br>R: CATAAAGTTGCCTTCTGCTCTCTCT
FLRL6	CUST_84985_PI42875355	chr1(91415981, 91459328, −)	F: CAGAGTTTGTGCGATGATGATTC<br>R: CTGGGTGAAGGTACGTTTGGTT
FLRL7	CUST_89632_PI428753551	chr19(10063631, 10064017, −)	F: ATGGCTGGGATTGGTACTTG<br>R:ATGGTGGGCATGAGAGAACT
FLRL8	CUST_94177_PI428753551	chr8(84001705, 84004770, +)	F: GACCAGCAGAGGATGATCTTGACT<br>R: TTGCCCTCCCTCGAACTTAA
